# Keel bone damage and associated physiological parameters in traditional chicken breeds and hybrid layers

**DOI:** 10.3389/fphys.2026.1973493

**Published:** 2026-10-20

**Authors:** Lisa Hildebrand, Saskia Neukirchen, Mareike Fellmin, Julia Mehlhorn, Stefanie Petow, Lars Schrader, Jenny Stracke, Steffen Weigend, Beryl Eusemann-Keller

**Affiliations:** 1 Institute of Animal Welfare and Animal Husbandry, Friedrich-Loeffler-Institut, Celle, Germany; 2 Institute for Animal Science – Farm Animal Ethology, University of Bonn, Bonn, Germany; 3 Poultry Research Center, Bruno-Dürigen Institute, Rommerskirchen, Germany; 4 Medical Faculty and University Hospital Düsseldorf, Institute for Anatomy I, Heinrich Heine University Düsseldorf, Düsseldorf, Germany; 5 Chair of Animal Welfare, Ethology, Animal Hygiene and Animal Husbandry, Department of Veterinary Sciences, Faculty of Veterinary Medicine, Ludwig-Maximilians-University, Munich, Germany; 6 Department of Animal Sciences, Animal Husbandry, University of Göttingen, Göttingen, Germany; 7 Institute of Farm Animal Genetics, Friedrich-Loeffler-Institut, Neustadt Mariensee, Germany; 8 Center for Integrated Breeding Research, University of Goettingen, Göttingen, Lower, Saxony, Germany; 9 Institute of Animal Hygiene and Veterinary Public Health, Faculty of Veterinary Medicine, Leipzig University, Leipzig, Germany

**Keywords:** estradiol, follicle-stimulating hormone, keel bone damage, keel bone fractures, laying hens, traditional breeds, x-ray

## Abstract

**Introduction:**

Keel bone damage is an important welfare issue in laying hens. Although some risk factors have been identified, the etiology is not fully clear. In previous studies, traditional breeds exhibited less keel bone fractures than hybrid layers.

**Aim:**

Thus, this study aimed at finding out more about potential influencing factors by comparing two traditional breeds, Rhode Island Red (RHO) and American Leghorn (AL), and the two different hybrid layers Lohmann Brown Classic (LB) and Lohmann Sandy (LSA) in terms of keel bone damage and potentially related parameters.

**Methods:**

The animals were kept in groups of 12 hens and one or two roosters, under natural daylight conditions, and with access to outdoor enclosures. Blood sampling was conducted at five and radiography at six time points between the 16^th^ and 72^nd^ week of age. The radiographs of the keel bones were evaluated for keel bone fractures, deviations, radiographic density, ossification, and length. Furthermore, the concentration of blood ionized calcium and plasma total calcium, phosphate, 17β-estradiol, and follicle-stimulating hormone (FSH) was measured.

**Results:**

The overall prevalence of keel bone fractures was significantly higher in LB and LSA (90.1% and 87.5%, respectively) compared to RHO (0%) and AL (9.1%). RHO were larger and started laying distinctly later than the other genotypes. In addition, prolonged weight gain and less progressed keel bone ossification in the 24^th^ week of age compared to the other genotypes indicate slower physical development. The results on radiographic density, ossification, and age at first egg indicate that ossification and possibly mineralization were less progressed in the hybrid layers than in AL at onset of lay. Plasma 17β-estradiol increased around the onset of lay, but did not significantly differ between the genotypes during the laying period. Furthermore, between the 33^rd^ and 52^nd^ week of age, there was a significant increase in plasma FSH in all genotypes, which may be attributable to seasonal changes in the length of daylight.

**Discussion/Conclusion:**

In summary, this study offers insights into keel bone health, bone traits, and reproductive parameters in different chicken genotypes, which may improve understanding and contribute to future strategies to reduce keel bone damage.

## 1 Introduction

Physical integrity is one of the cornerstones of animal welfare (Farm Animal Welfare in Great Britain: Past, Present and Future, 2009). In laying hens, skin disorders, soft tissue and integument damage, and bone lesions have been identified as some of the most relevant welfare concerns (Nielsen et al., 2023). Besides occasional wing and leg fractures, keel bone damage is the most frequently observed bone lesion in laying hens (Sandilands et al., 2009). The term “damage” includes two different types of lesions: keel bone fractures and keel bone deviations. Keel bone fractures are complete or incomplete breaks of the keel bone. In practice, they are often identified through the formation of repair tissue – the callus (Casey-Trott et al., 2015). The majority of keel bone fractures are located in the caudal part of the keel bone (Casey-Trott et al., 2017; Habig et al., 2021a; Sallam et al., 2024). Older laying hens often show more than one callus, indicating multiple fractures (Emmert et al., 2024; Thøfner et al., 2021). In the animal, keel bone fractures lead to reduced mobility and changes in behavior, which indicate that the animals experience pain and stress (Armstrong et al., 2020; Montalcini et al., 2024; Nasr et al., 2012). Furthermore, in some studies, affected hens showed a reduced laying performance and higher feed and water consumption, which brings an additional, economic dimension to the issue (Nasr et al., 2013; Rufener et al., 2019; Wei et al., 2020). Keel bone deviations are defined as changes in the shape of the keel bone, often shown as bend, S-shape, indentation, or a combination of these (Casey-Trott et al., 2015). The consequences are not as well-investigated, but it has been suggested that especially severe deviations can also lead to pain and restriction in mobility (as reviewed by Riber et al., 2018). Both keel bone fractures and deviations are common in laying hens, with prevalence rates reported between 30 and 100% of assessed hens within a flock (Casey-Trott et al., 2017; Eusemann et al., 2018; Sallam et al., 2025; Thøfner et al., 2021; Wilkins et al., 2011).

The prevention and reduction of keel bone damage in laying hens is especially difficult because of its multifactorial genesis. Early studies have linked keel bone deviations to genetics, the lack of vitamin D during the winter, and early access to perches (Warren, 1937). Housing system and barn interior (e.g., ramps, perches) can have an impact on the risk for high-impact incidents that lead to keel bone fractures (Käppeli et al., 2011; Stratmann et al., 2015). However, the lack of soft tissue damage and the morphology of some fractures suggest that they do not only occur due to high-impact falls and collisions, but as a result of insufficient bone stability (Gretarsson et al., 2024; Thøfner et al., 2020).

While keel bone deviations are found in animals of both sexes, fractures are seen predominantly in hens (Kittelsen et al., 2021; Thøfner et al., 2021). The strong connection with laying activity has also been demonstrated in a study by (Eusemann et al., 2020). The risk of developing a keel bone fracture until the 61^st^ week of age (woa) was reduced by 80% in hens that were prevented from laying compared to control hens. There are different ways in which egg-laying, especially in modern laying hens, may interfere with skeletal stability and increase the susceptibility for keel bone fractures. To begin with, it is important to note that both bone substance and eggshell include a substantial proportion of calcium (Farmer et al., 1986; Gebhardt-Henrich et al., 2017). Therefore, they stand in competition with each other for the available calcium. Thus, consistently high egg production could lead to demineralization or loss of bone substance over time. Another possible contributing factor is a change in the hormone-controlled bone and calcium metabolism during the laying period that persists over multiple months in modern laying hens. 17β-estradiol is the most abundant form of estrogen and is secreted by small follicles during the laying period (as reviewed by Hanlon et al., 2022b). The growth of the follicles and secretion of estradiol is promoted by follicle-stimulating hormone (FSH), which originates from the pituitary gland (as reviewed by Mans and Taylor, 2008). In the bone, 17β-estradiol induces the formation of medullary bone, a special type of bone that supplies calcium during the formation of the eggshell (Squire et al., 2017; Whitehead, 2004). Due to its structure, medullary bone does not contribute much to bone stability, compared to cortical or trabecular bone (Whitehead, 2004; Wilson and Duff, 1990). However, in some cases, the formation of medullary bone has been linked to the loss of structural bone (Wilson and Thorp, 1998). In modern laying hens, a further aggravating factor is the early onset of lay (Gebhardt-Henrich and Fröhlich, 2015; Kittelsen et al., 2020; Thøfner et al., 2021). When the animal hatches, the keel bone is not yet fully ossified (Buckner et al., 1948). Ossification proceeds from cranial to caudal and, according to different sources, is completed between 25 and 40 woa depending on the genotype (Buckner et al., 1948; Gretarsson et al., 2024). Therefore, when a laying hen starts laying around 20 woa, ossification of the caudal part of the keel bone still has to take place, while at the same time medullary bone is built and calcium is needed for the formation of the eggshell. Additionally, the newly built bone is still flexible, which might make it more prone to greenstick fractures, as suggested by Casey-Trott et al. (2017) and Thøfner et al. (2020).

Besides egg-laying and onset of lay, genetically controlled or influenced traits can have an effect on the development of keel bone damage. The genetic influence often becomes visible when comparing white and brown layers, as there is a relatively great phylogenetic distance between them and they descend from different breeds (Malomane et al., 2019). While white layers descend from White Leghorn lines, brown layers originate from Rhode Island Red lines and White Plymouth Rock (Fulton, 2006). They show differences in fear response, space usage, and spatial skills as well as bone properties, which may all influence the risk for fractures (Adler et al., 2024; DePaoli et al., 2024; Garant et al., 2023; Jansen et al., 2020; Rentsch et al., 2023). In some studies, differences in the prevalence of keel bone damage have been observed between white and brown layers (Eusemann et al., 2018; Fawcett et al., 2020; Gebhardt-Henrich and Fröhlich, 2015; Rufener et al., 2019). However, across different studies, there is no consistent trend of one of them experiencing more keel bone fractures.

Although some internal factors for the development of keel bone damage have been identified, pathogenesis and etiology are still not fully clear. Investigating chicken genotypes that are not bred on a commercial level not only provides a broader overview of the extent of this welfare relevant issue, comparing the different genotypes also offers the opportunity to learn more about hen-related influencing factors and increase the knowledge on changes that have occurred under the selection for egg production traits, which may have led to an increased susceptibility to fractures. In a previous study (Hildebrand et al., 2026), we have already compared a hybrid layer (Lohmann Selected Leghorn) and two low-performing genotypes (Junglefowl phenotype and Sumatra) with an annual laying performance of less than 100 eggs. In this study, fractures were only found in LSL, but not in the low-performing genotypes. Furthermore, the results indicated that keel bone ossification and mineralization are less progressed in LSL than in Junglefowl phenotype at onset of lay. The aim of this follow-up study was to investigate two phylogenetically divergent traditional breeds (Rhode Island Red (RHO) and American Leghorn (AL)) with a moderate laying performance as well as two high performing hybrid layers (Lohmann Brown Classic (LB) and Lohmann Sandy (LSA)) in terms of keel bone damage and associated parameters.

Due to previous findings, we hypothesized that the two hybrid layers would show more keel bone fractures than the medium performing layers. Furthermore, we hypothesized that the earlier and higher laying activity in the hybrids would be reflected by higher plasma levels of reproductive hormones and total calcium.

## 2 Animals, materials, and methods

The animal experiment was approved by the State Agency for Nature, Environment and Consumer Protection of North Rhine-Westphalia (Reference number: 81-02.04.40.2023.VG039).

### 2.1 Animals

RHO is a brown-laying, traditional breed that descends from Rhode Island local chickens, yellow Cochins, and brown-red Malays (Schmidt, 1999). AL are white Leghorns that originate from Italian local breeds, which were exported to the US and bred for higher laying performance. Their feathers are white and they lay eggs with white shells (Schmidt, 1999). According to the breeding company, the hybrids LB and LSA produce over 320 eggs/hen during a 72 weeks production period and lay eggs with brown and cream-colored shells, respectively (LOHMANN BREEDERS GmbH, 2021a, LOHMANN BREEDERS GmbH, 2021b).

There was one group per genotype. The final experimental groups were selected according to phenotypic breed standards and healthy appearance and consisted of 12 hens accompanied by two roosters. The number of animals was determined by housing capacity. The animals hatched in the facilities of the Scientific Poultry Research Center in Rommerskirchen (Germany) in mid-April, which corresponds to spring. The hatching eggs for LB and LSA were obtained from Lohmann Deutschland GmbH & Co. KG (Ankum, Germany). The hatching eggs for the other genotypes were either produced on site (4 out of 12 AL hens) or provided by different breeders that collaborate with the Scientific Poultry Research Center. The eggs were incubated in artificial brooders (Favorit-Olymp Series, HEKA Brutgeräte GmbH & Co. KG, Rietberg, Germany). Until the 18^th^ day of incubation, the eggs were kept at 37.8 °C and 53% humidity and tilted three times in 120 minutes. Pre-hatching (from the 19^th^ day onward) was carried out at 37.5 °C with humidity increasing to 63-80%. Hatching rate was lowest in AL (70.6%) and highest in LSA (88.9%).

The groups were kept separate from each other. Housing conditions did not resemble commercial settings but were comparable to a previous study about different genotypes (Hildebrand et al., 2026) to facilitate comparisons. The animals were reared in 4.3 m^2^ indoor compartments equipped with brooder plates, infrared and radiant heaters, and a total of five perches at 12 cm, 30 cm, 50 cm, 70 cm, and 90 cm height, with 2m length each and a rhomboid cross section of 2.5 cm x 4 cm. Feed and water were provided through feeding troughs and bell drinkers. From three woa onward, the groups had access to a 5.9–7 m^2^ outdoor area. For enrichment, the animals were provided with litter, straw bales, hay nets, and loose feed on a rotational basis.

In the sixth woa, each group was moved to a large outdoor enclosure (125 m^2–^205 m^2^) with different natural and manmade enrichment elements and a chicken house. The chicken house had a floor area of 5.1 m^2^ and was equipped with a 4 cm x 6 cm rectangular, 2.8 m long perch that was placed at approximately 90 cm height just above a dropping board. Furthermore, each group was provided with two nests on floor level. The nests had a floor area of 40 cm x 45 cm. There was no artificial lighting program. The photoperiod therefore corresponded to the natural daylight that passed through the windows (total window area: 1.7 m^2^) and varied between 7:54 h and 16:33 h per day. Feed and water were provided ad libitium through 34 cm diameter bell drinkers and round feeders with a diameter of 40 cm.

Due to management reasons, the animals were relocated to the facilities of the University of Bonn in the 46^th^ woa. Here, the groups were kept in 5.3 m^2^ indoor compartments with an outdoor run between 8.6 m^2^ and 10.8 m^2^. The indoor compartments were equipped with three 4 cm x 6 cm rectangular, 2 m long perches placed at 24 cm and 50 cm height and a EUROPA nest unit with three single nests (Landmesser, Erkelenz, Germany; distributed by Siepmann GmbH, Herdeckel, Germany), which was placed on floor level. Hay nets, tree branches, pecking stones, and hanging devices for fresh feed, which was provided twice a week, were included for enrichment.

The feed was obtained from Deutsche Tiernahrung Cremer GmbH & Co. KG (Düsseldorf, Germany). Until 7 woa, the animals received a complete feed for chicks (All-Mash A: 11.4 MJ ME/kg, 18% CP, 1% Ca, 0.6% P). Between 7 woa and onset of lay, they were fed a complete feed for pullets (All-Mash R: 11.4 MJ ME/kg, 14.5% CP, 0.9% Ca, 0.5% P) and after onset of lay, they received a complete feed for laying hens (VoMiGo LAF: 11.2 MJ ME/kg, 17% CP, 3.6% Ca, 0.5% P). After the relocation in the 46^th^ woa, feed was changed from pellets to meal to provide extra activity.

A total of eight hens dropped out of the experiment due to predation (1 LB and 4 LSA), death (1 RHO and 1 AL), or medical condition (1 RHO), respectively.

### 2.2 Laying performance

Laying performance was assessed at group level. For each group, the age at first egg and the age at 10% laying performance were recorded. After reaching 10% group laying performance, the number of eggs was counted for one year and used to calculate the average egg number per hen and year. Additionally, for each group, laying intensity was calculated for each calendar month to investigate seasonal fluctuation.

### 2.3 Radiography, blood sampling and ultrasonography

Similarly to the previous study (Hildebrand et al., 2026), radiography, ultrasonography, and blood sampling were conducted at five different time points before and during the laying period: in the 16^th^, 24^th^, 33^rd^, 52^nd^, and 72^nd^ woa. A further radiographic examination took place in the 47^th^ woa to assess if the relocation of the birds from the Scientific Poultry Research Center to the facilities of the University of Bonn had led to new fractures. Additionally, body weight was recorded as part of the data collection, using a hanging scale (Kern and Sohn GmbH, Balingen, Germany) or a table scale (Albert Kerbl GmbH, Buchbach, Germany; only for RHO in the 72^nd^ woa to reduce the strain caused by their higher body weight).

#### 2.3.1 Radiography

For the radiographic examination, the animals were positioned as described in Hildebrand et al. (2024). Radiographs were taken at 50 kV and 2.5 mAs. For further evaluation, a home-built aluminum step wedge with 18 steps between 0.5 and 4.75 mm height was included in every image.

The evaluation of the radiographs was conducted with the software ImageJ (Version 1.48, Rasband, W., National Institute of Health, Bethesda, Maryland, United States) as previously described by Hildebrand et al. (2026) (**Figure 1**). For the assessment of fractures, a customized ImageJ tool was used to subdivide the keel bone into equal thirds, as proposed by Baur et al. (2020), but without the labeling of the apices. For each third, the number of fractures with and without callus formation was scored separately on a three level scale (0 = no fracture, 1 = one fracture, 2 = two or more fractures). Fractures without callus formation were defined as radiolucent gaps or lines in the structure of the keel bone. Fractures with callus formation were visible as more radiodense spots with irregular margins. In four cases (2 AL in the 24^th^ woa and 2 RHO in the 33^rd^ woa), the keel bone and the tibiotarsus strongly overlapped. As this can interfere with the identification of keel bone fractures, the respective images were not evaluated for fractures.

**Figure 1:**
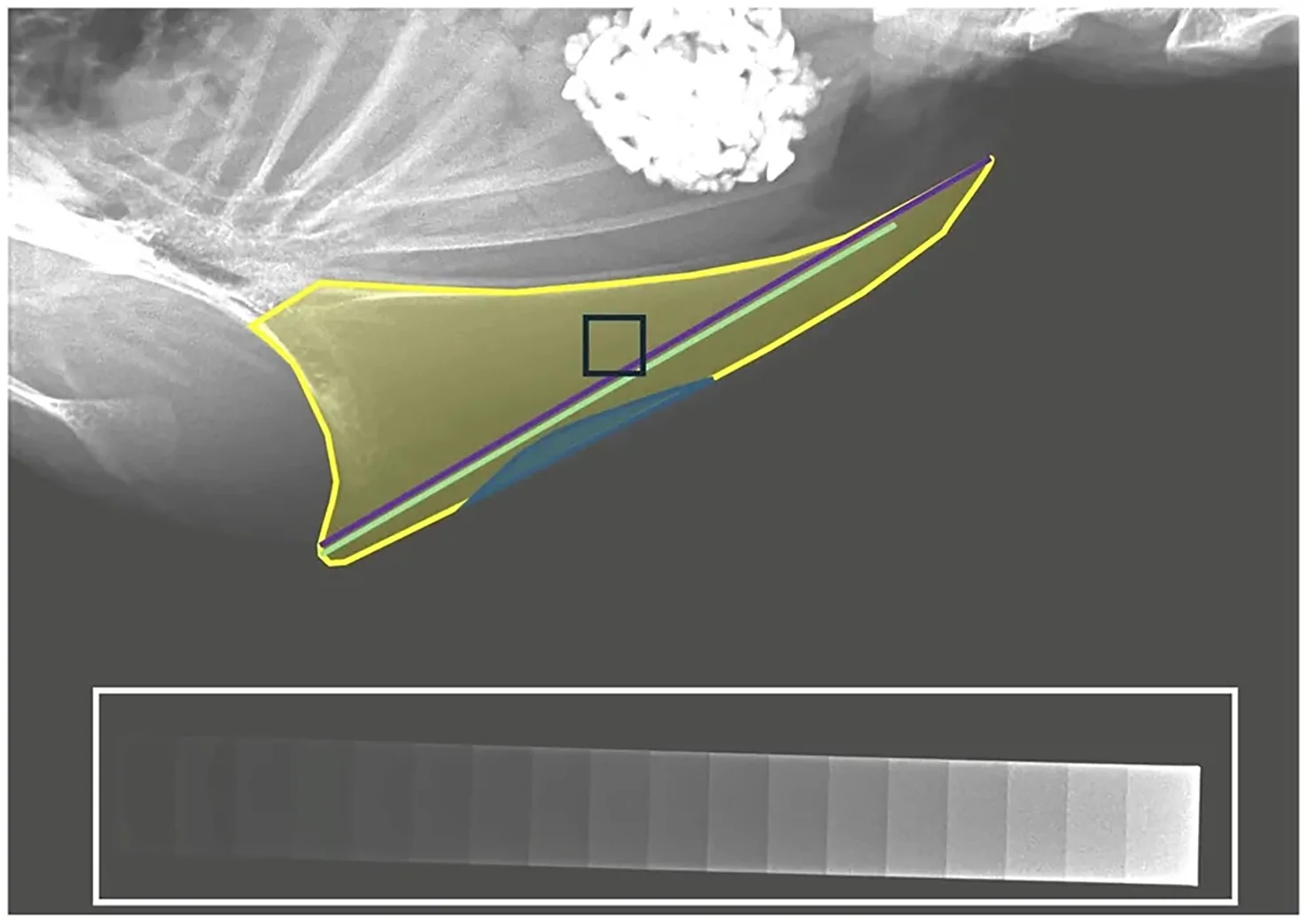
Keel bone measures obtained from the X-ray images: Total length (purple), length of the ossified part (light green), lateral surface area (yellow), area of deviation (blue), and radiographic density in a square placed in the middle of the keel bone (black) using an aluminum step wedge (white). Reproduced from Hildebrand et al. (2026).

Keel bone length, the length of the ossified part of the keel bone, lateral surface area, and area of deviation were measured using the “Length” and the “Polygon” tools in ImageJ, respectively. The degree of ossification was calculated by dividing the length of the ossified part of the keel bone by the full keel bone length. In order to estimate the severity of keel bone deviations, the percentage of deviated keel bone area (POD) was calculated by dividing the area of deviation by the lateral keel bone surface area. In two cases, the deviation could not clearly be separated from changes in keel bone shape due to a fracture. The respective POD values were therefore excluded from the analysis. As a proxy for bone mineral density, the radiographic density of the keel bone was measured. As described by Eusemann et al. (2020), the aluminum step wedge was used to create a standard curve and calculate the gray values of the image in mm aluminum equivalent. Radiographic density was then measured in a square, which was placed in the middle of the keel bone using a customized ImageJ tool, as validated in Hildebrand et al. (2026).

#### 2.3.2 Determination of blood parameters

Blood collection took place between 11 a.m. and 3 p.m. The samples were drawn from the ulnar vein using lithium heparin microtubes or Monovettes (both SARSTEDT AG & Co. KG, Nuernbrecht, Germany). Ionized calcium was measured on site. For the measurement of the remaining parameters, the blood samples were cooled down on crushed ice, centrifuged at 4600 rpm and 4–10 C for 10 minutes with the Thermo Scientific™ Heraeus™ Fresco centrifuge (Thermo Fisher Scientific, Waltham, Massachusetts), and transported to facilities of the Friedrich-Loeffler-Institut. In the 33^rd^ woa, two LB plasma samples were accidentally pooled. Due to the relatively low animal number in the study, the values obtained from the pooled sample were used for both animals.

Ionized calcium was measured directly after sampling. To this aim, 300 µL of blood were taken from the tube and placed on the electrode of the LAQUATwin-Ca-11C (Horiba Ltd., Kyoto, Japan). The portable device was recalibrated every ten measurements. Calcium and phosphate were measured with the Indiko™ Clinical Chemistry Analyzer (Thermo Fisher Scientific, Waltham, Massachusetts). Estradiol and FSH were determined using commercially available ELISA kits (ABIN RE52041, IBL International GmbH, Hamburg, Germany and ABIN 6966858, antibodies-online GmbH, Aachen, Germany). The measurements were conducted in duplicate and repeated if the coefficient of variation exceeded 15% and the standard deviation was higher than 10. For the measurement of 17β-estradiol, the samples were diluted 1:3. Due to the wide range of FSH concentrations, not all samples could be measured in the same dilution. Initially, all samples were diluted 1:6. However, higher dilutions were chosen whenever the concentration of the sample exceeded the measuring range of the ELISA at the first measurement attempt.

#### 2.3.3 Ultrasonography

Ultrasonography was conducted with the ultrasound scanner CHISON SonoBook 6 Portable Ultrasound (CHISON Medical Technologies Co., Wuxi, Jiangsu, China). To determine the follicle status, the animal was positioned on its right side and the left leg was slightly pulled cranial so that the examiner could place the transducer caudal from the ribs. If dominant follicles (> 10 mm) were visible, the animal was considered as *in-lay*. If no dominant follicles were visible, the animal was considered as *non-laying*. However, false negative findings are possible. Therefore, additionally, the radiographs were assessed to detect calcified eggs. In one case, no follicles were found during the ultrasonography, but an egg was visible on the radiograph. Thus, the respective animal was also considered as *in-lay*.

After the sonography of the ovaries, the pectoral muscle thickness was measured as described in Hildebrand et al. (2026). The left leg was moved back and the transducer was placed cranial on the breast muscle and moved caudal until *carina* and *corpus sterni* were visible. Then, the distance between the skin and the junction between *corpus* and *carina sterni* was measured.

### 2.4 Statistical analysis

The statistical analyses were performed using the software RStudio (Version 4.5.1, Posit PBC, Boston, Massachusetts, USA). P-values < 0.05 were considered statistically significant. Additionally, due to the exploratory nature of this study, p-values between 0.05 and 0.1 were noted and referred to as approaching significance, trend, or tendency. Levene’s tests were used to check homoscedasticity and QQ-plots, histograms, and scatter plots were used to assess residual distribution.

#### 2.4.1 Fractures and deviations

In order to assess the prevalence rates of keel bone fractures and deviations, both POD and fracture scores were converted into simplified binary variables as follows: For fractures, 0 indicates no visible fracture or callus in any section of the keel bone and 1 indicates at least one visible fracture or callus in any section of the keel bone. For deviations, the binary variable was set to 0 if POD was 0 and to 1 if POD was higher than 0, i.e., if a deviation was measurable in the radiograph. Subsequently, for each age and genotype combination, fracture and deviation prevalence rates were calculated using exact binomial Clopper-Pearson intervals. Additionally, a bias-reduced generalized linear model (brglm, Firth-Method) with genotype as fixed effect was used to assess overall fracture prevalence. For the former, RHO were chosen as reference group since they did not show any fractures. All animals that were investigated until the end of the study without missing values due to overlapping were included in this analysis. *Post hoc* tests were conducted using estimated marginal means with Tukey-adjusted p-values. To assess whether deviation severity differed between the genotypes, POD of animals with visible deviation (POD > 0) was investigated in a linear mixed model with genotype as fixed and animal as random effect. Since not all genotypes showed deviations at all time points, age was not included as fixed effect. P-values were obtained using the anova() function and Tukey tests were performed for *post hoc* testing.

#### 2.4.2 Continuous keel bone and blood parameters

Linear mixed models with age, genotype, and their interaction as fixed effects were built for body weight (log-transformed), keel bone length, pectoral muscle thickness, radiographic density, total calcium, ionized calcium, phosphate, 17β-estradiol (log-transformed), and FSH (log-transformed). To consider the repeated measurements, the animal was included as a random effect. For the analysis of the reproductive hormones, outliers (< Q1 - 1.5*IQR or > Q3 + 1.5*IQR) were removed from the dataset. For total calcium, phosphate, and FSH, varIdent variance structures were used to model heterogeneous variances in linear mixed models. Due to findings in the previous study (Hildebrand et al., 2026), an additional linear mixed model with follicle status, genotype, and their interaction as fixed effects and the animal as a random effect was built for total calcium (log-transformed). As wrong negative findings are possible for follicle status, strong upper outliers (Q3 + 3.5*IQR, i.e., 4.37 mmol/l) were removed from the dataset for this purpose. For the analysis of keel bone ossification, only the first three time points (until 33 woa) were included, as ossification was completed in all genotypes afterward. Since the data did not meet the criteria for parametric testing, an aligned ranks transformation ANOVA was performed. *Post hoc* pairwise comparisons were made using Tukey tests.

## 3 Results

To improve readability, not all p-values for non-significant *post hoc* comparisons are reported in the results section. All p-values as well as the estimated differences between the groups are shown in **Supplementary Material A**.

### 3.1 Body weight

The interaction between age and genotype showed a significant influence on body weight (F(12, 162) = 19.25, p < 0.001) (**Figure 2**). In RHO, body weight significantly increased between the 16^th^ and 24^th^ woa, between the 24^th^ and 33^rd^ woa, and between the 33^rd^ and 52^nd^ woa (p < 0.001). AL and LSA showed increases in body weight between the 16^th^ and 24^th^ woa and the 24^th^ and 33^rd^ woa (AL: p < 0.001, LSA: 16^th^-24^th^ woa: p < 0.001; 24^th^-33^rd^ woa: p = 0.006). In LB, significant increases in body weight were observed between the 16^th^ and 24^th^ woa and between the 33^rd^ and 52^nd^ woa (p < 0.001). Throughout the study, RHO had a significantly higher body weight than the other three genotypes (p < 0.001). Additionally, in the 16^th^ and 24^th^ woa, LB and LSA were significantly heavier than AL (16^th^ woa: p = 0.002, 24^th^ woa: AL-LB: p < 0.001, AL-LSA: p = 0.004). Mean body weight over the last two sampling periods, when no significant changes were observed in any genotype, was 3263 ± 253 g in RHO, 2117 ± 151 g in AL, 2151 ± 115 g in LB, and 2071 ± 75 g in LSA.

**Figure 2:**
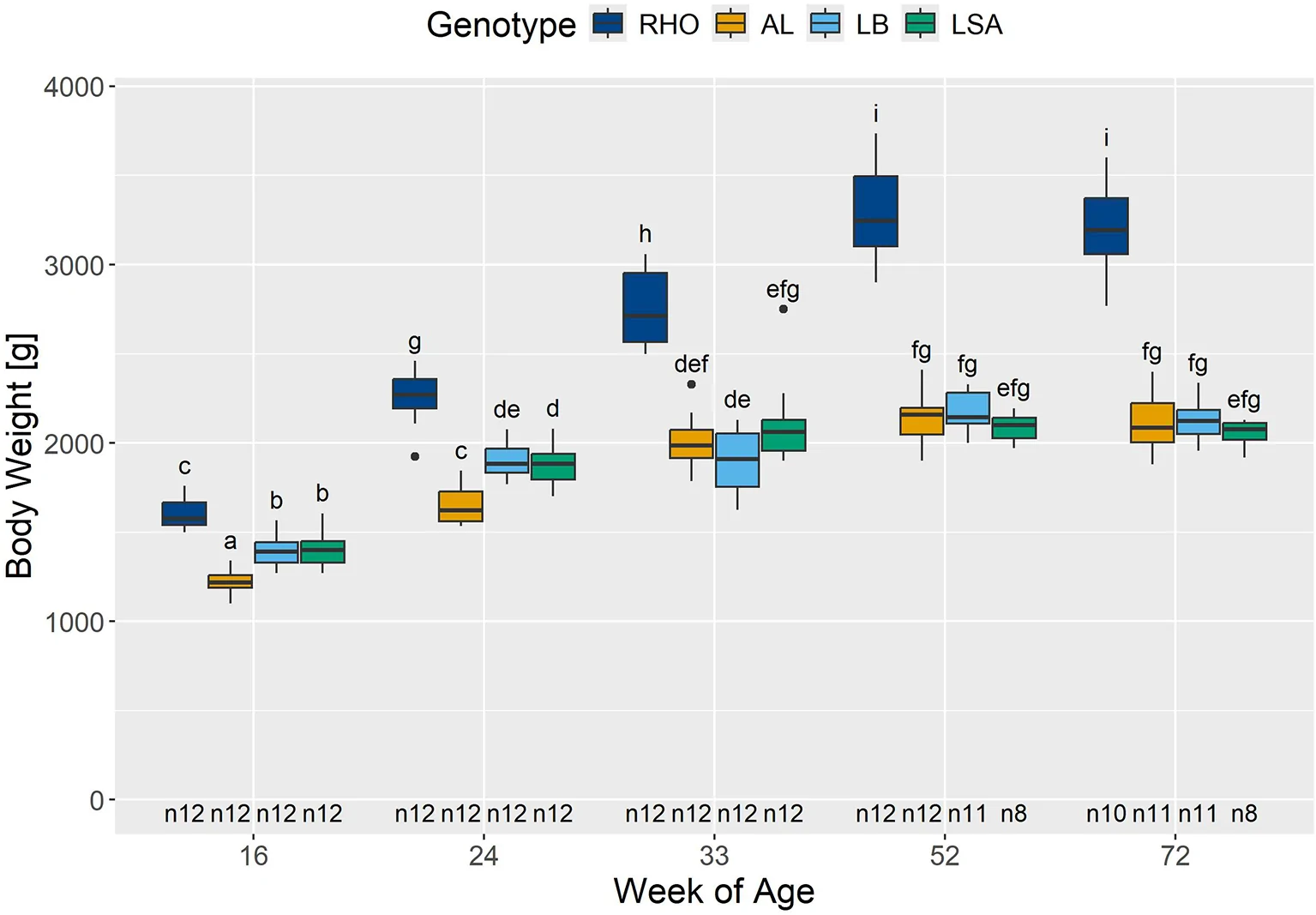
Body weight in relation to age and genotype. The x-axis represents age and the y-axis shows body weight in gram. Different genotypes are illustrated by different colors (see legend). Significant differences (Tukey-Kramer, p < 0.05) are indicated by no common subscript letter. The number of animals can be seen below the boxes of the boxplot (e.g., n12 indicates 12 animals).

### 3.2 Onset of lay and laying performance

The age at onset of lay, given as both age at first egg as well as age at 10% group laying performance, differed between the genotypes (**Table 1**). LB and LSA started egg-laying first, followed by AL and then RHO.

**Table 1:** Age at first egg and 10% group laying performance.

Genotype	Age at first egg [week of age]	Age at 10% group laying performance [week of age]
RHO	30	32
AL	23	28
LB	18	18
LSA	19	20

Average laying performance during the first laying year was 165 eggs/hen in RHO, 142 eggs/hen in AL, 318 eggs/hen in LB, and 327 eggs/hen in LSA. With laying intensities varying from 27% to 90% and from 0% to 66%, respectively, RHO and AL exhibited greater fluctuations between the calendar months than the hybrid layers, that had laying intensities between 61% and 97% (LB) and between 69% and 95% (LSA) (**Figure 3**). AL reached laying peak in March, while RHO reached peak performance in July.

**Figure 3:**
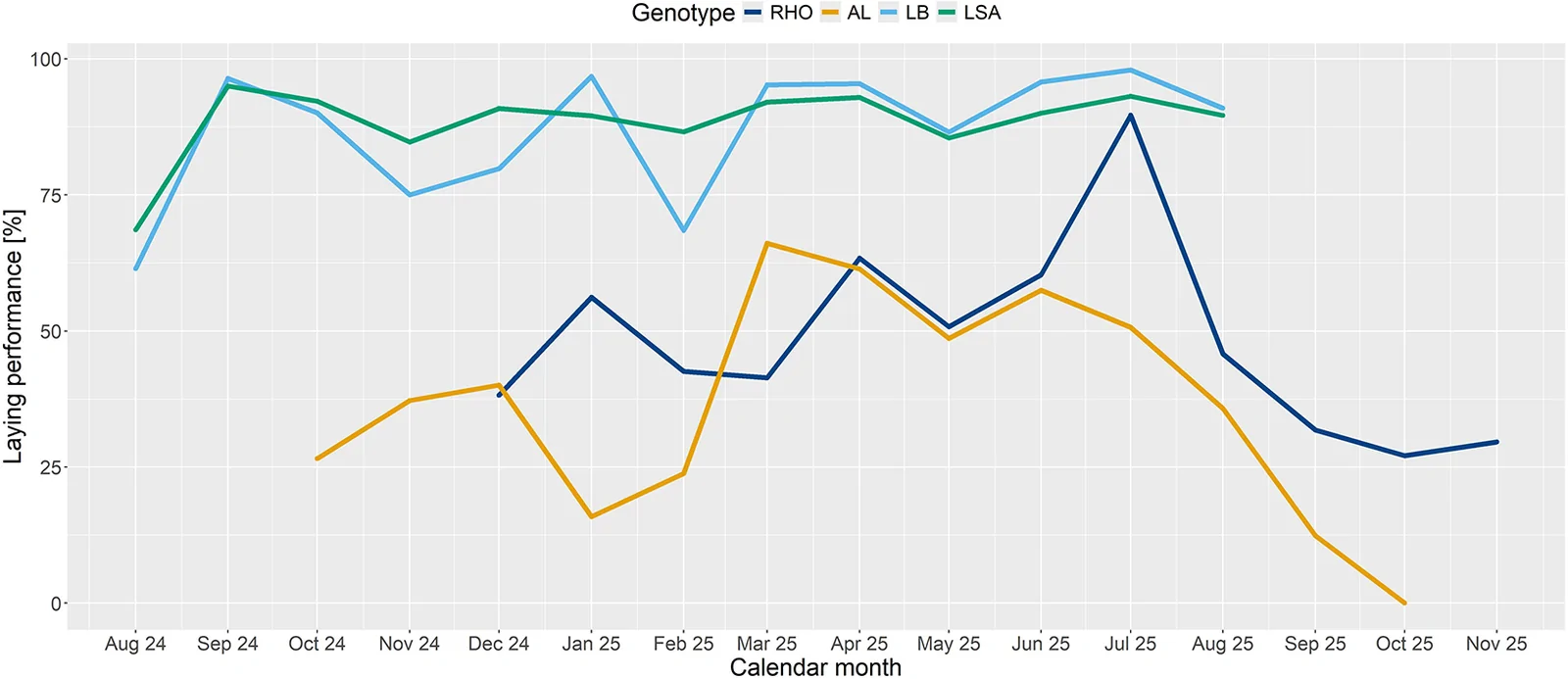
Mean laying intensity per calendar month across the different genotypes. The x-axis shows the respective calendar month and the y-axis the mean monthly laying performance. The different genotypes are indicated by different colors (see legend). For illustration purposes, the individual data points in this figure were connected.

### 3.3 Keel bone damage

No keel bone fractures were observed in the 16^th^ woa in any of the genotypes (**Figure 4**, **Table 2**). From the 24^th^ woa onward, prevalence rates increased with age. Overall prevalence rates, i.e., prevalence throughout the study, were 0% in RHO, 9.1% in AL, 90.1% in LB, and 87.5% in LSA. Compared to the reference group (RHO), LB and LSA showed significant differences in overall fracture prevalence (β = 4.99, p = 0.005 and β = 4.65, p = 0.009, respectively). Pairwise comparisons revealed that significantly more LB and LSA hens showed keel bone fractures compared to RHO and AL throughout the study (LB-RHO: p = 0.025, LB-AL: p = 0.014, LSA-RHO: p = 0.046, LSA-AL: p = 0.035). In contrast, there were no significant differences between LB and LSA (p = 0.994) or RHO and AL (p = 0.926). Out of 47 radiographs with fractured keel bones, 11 displayed at least one visible fracture gap (**Figure 4**). In all other cases, fractures were visible through callus formation, not fracture gaps. In 42 out of the 47 radiographs, fractures were located in the caudal third of the keel bone, and in 9 out of 47, the middle section was affected. Throughout the study, no fractures were found in the cranial third of the keel bone.

**Figure 4:**
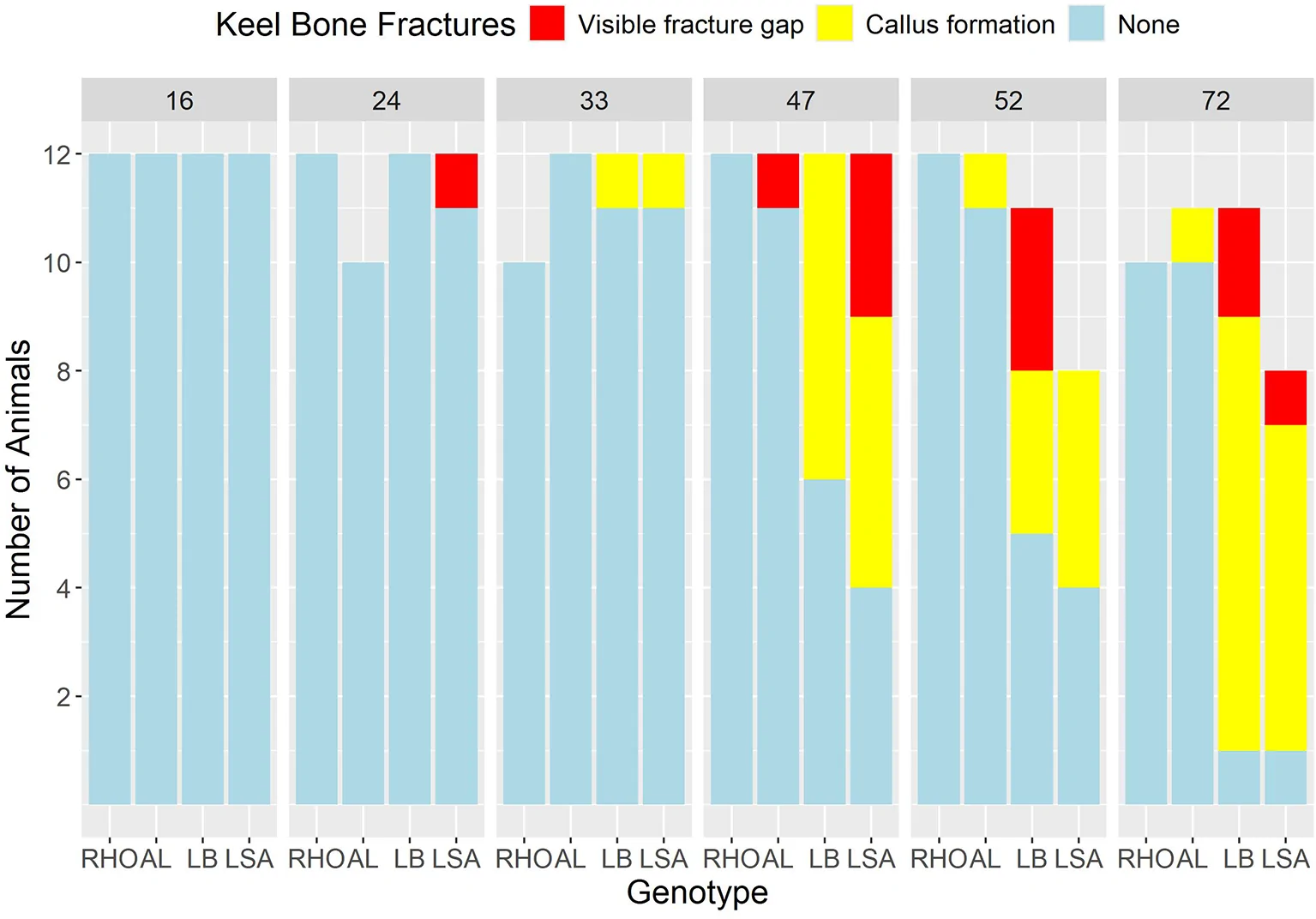
Number of animals with and without keel bone fractures in relation to age and genotype. Light blue represents animals without any visible sign of keel bone fractures, yellow represents animals with callus formation and red represents animals with at least one visible fracture gap.

**Table 2:** Prevalence of keel bone fractures in the different genotypes throughout the study.

Genotype	16^th^ woa	24^th^ woa	33^rd^ woa	47^th^ woa	52^nd^ woa	72^nd^ woa	Overall
RHO	0 [0 - 26.5]	0 [0 - 26.5]	0 [0 - 30.8]	0 [0 - 26.5]	0 [0 - 26.5]	0 [0 - 30.8]	0 [0 - 30.8]
AL	0 [0 - 26.5]	0 [0 - 30.8]	0 [0 - 26.5]	8.3 [0.2 - 38.5]	8.3 [0.2 - 38.5]	9.1 [0.2 - 41.3]	9.1 [0.2 - 41.3]
LB	0 [0 - 26.5]	0 [0 - 26.5]	8.3 [0.2 - 38.5]	50 [21.1 - 78.9]	54.5 [23.4 - 83.3]	90.1 [58.7 - 99.8]	90.1 [58.7 - 99.8]
LSA	0 [0 - 26.5]	8.3 [0.2 - 38.5]	8.3 [0.2 - 38.5]	66.7 [34.9 - 90.1]	50 [15.7 - 84.3]	87.5 [47.3 - 99.7]	87.5 [47.3 – 99.7]

Prevalence rates are given in percent with the 95% exact clopper pearson intervals in square brackets underneath.

Prevalence rates of keel bone deviations fluctuated throughout the study (**Table 3**). In RHO, 25% of the hens showed keel bone deviations in the 16^th^ and 10% in the 72^nd^ woa, but no deviations were found in between. 8.3% of AL showed a keel bone deviation in the 52^nd^ woa, but no deviations were observed before or afterwards. In LB, deviations were present in the 33^rd^, 47^th^, 52^nd^, and 72^nd^ woa (8.3%, 25%, 18.2%, and 36.4%, respectively). LSA showed keel bone deviations with prevalences ranging between 25% (16^th^ and 24^th^ woa) and 81.8% (52^nd^ woa).

**Table 3:** Prevalence of keel bone deviations.

Genotype	16^th^ woa	24^th^ woa	33^rd^ woa	47^th^ woa	52^nd^ woa	72^nd^ woa
RHO	25 [5.5 - 57.2]	0 [0 - 26.5]	0 [0 - 26.5]	0 [0 - 26.5]	0 [0 - 26.5]	10 [0.3 - 44.5]
AL	0 [0 - 26.5]	0 [0 - 26.5]	0 [0 - 26.5]	0 [0 - 26.5]	8.3 [0.2 - 38.5]	0 [0 - 28.5]
LB	0 [0 - 26.5]	0 [0 - 26.5]	8.3 [0.2 - 38.5]	25 [5.5 - 57.2]	18.2 [2.3 - 51.8]	36.4 [10.9 - 69.2]
LSA	25 [5.5 - 57.2]	25 [5.5 - 57.2]	72.7 [39 - 94]	81.8 [48.2 - 97.7]	50 [15.7 - 84.3]	62.5 [24.5 - 91.5]

Prevalence rates are given in percent with the 95% exact clopper pearson intervals in square brackets underneath.

Within the group of animals with keel bone deviation, the genotype did not significantly influence POD (F(3, 16) = 1.26, p = 0.322) (**Figure 5**). Maximum POD observed throughout the study was 2.04% (LSA, 47^th^ woa).

**Figure 5:**
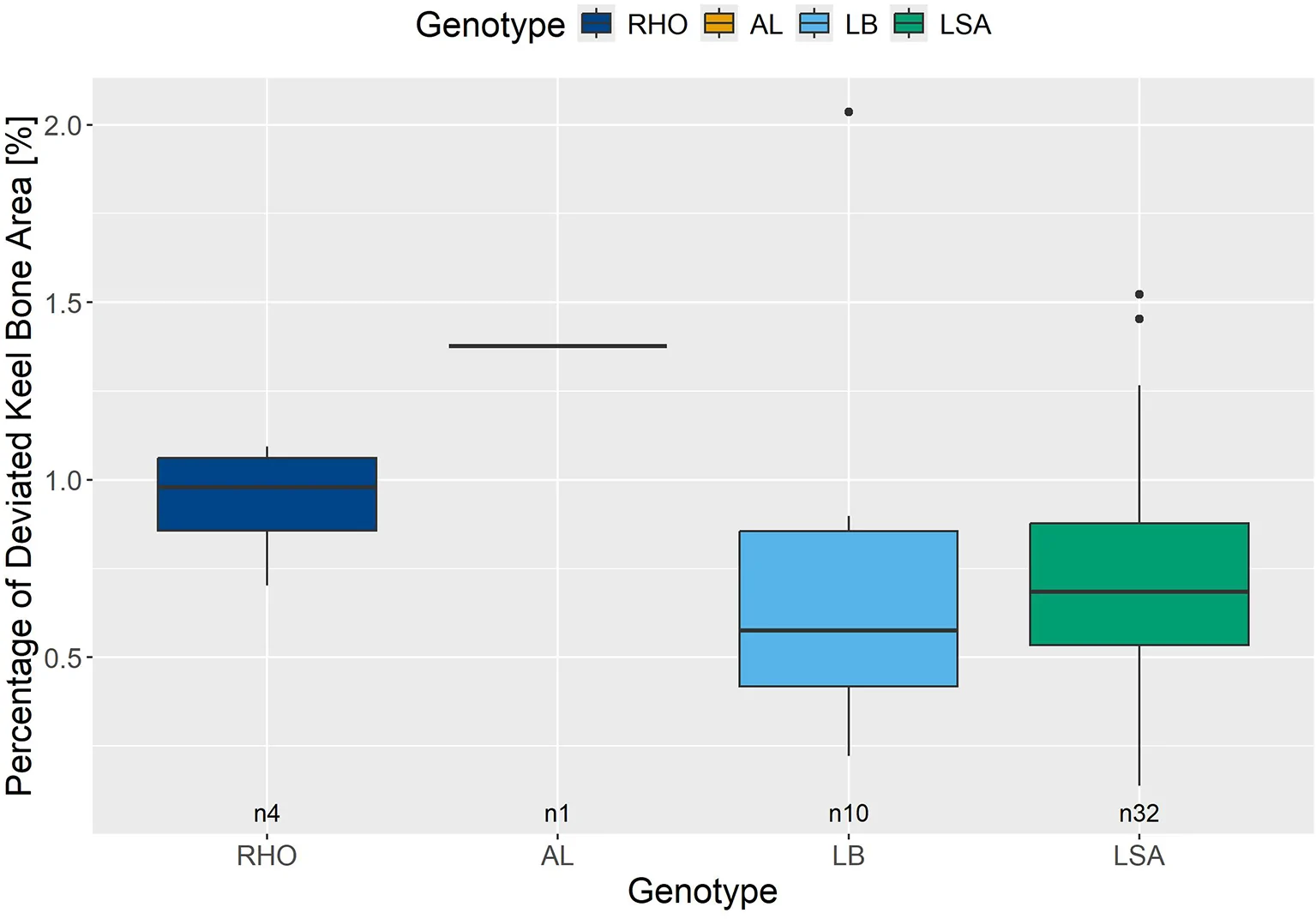
Percentage of deviated keel bone area across the hens with keel bone deviation (POD > 0%) in relation to genotype. The y-axis shows POD in %. Different genotypes are illustrated by different colors (see legend). There was no significant effect of genotype on POD. The number of radiographs included in the analysis can be seen below the boxes of the boxplot (e.g., n4 indicates 4 radiographs with measurable deviation).

### 3.4 Keel bone length and ossification

Keel bone length was significantly influenced by the interaction between genotype and age (F(15,207) = 9.31, p < 0.001) (**Figure 6**). Between the 16^th^ and 24^th^ woa, the keel bone length increased in all four genotypes (p < 0.001). Moreover, a significant decrease in the keel bone length of RHO was observed between the 24^th^ and 47^th^ woa (p = 0.004). In the 16^th^ woa, RHO had significantly longer keel bones than AL (p = 0.022) and tended to have longer keel bones than LB (p = 0.070). From the 24^th^ woa onward, RHO had significantly longer keel bones than the other three genotypes (p < 0.001). Furthermore, AL had significantly longer keel bones than LB in the 24^th^ woa (p = 0.007) and tended to have longer keel bones than LB in the 33^rd^ woa (p = 0.057).

**Figure 6:**
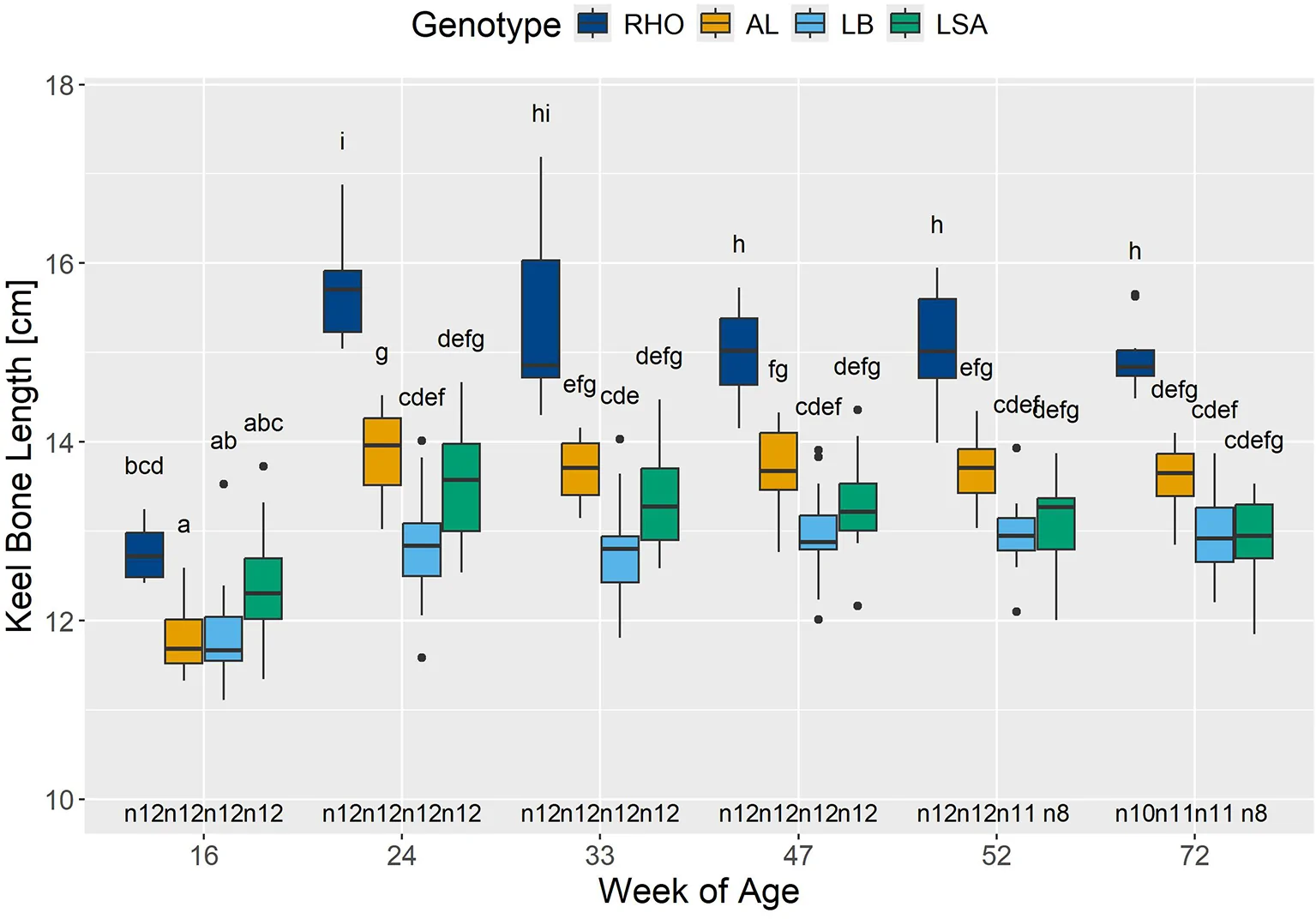
Keel bone length in relation to age and genotype. The x-axis represents age and the y-axis shows keel bone length in cm. Different genotypes are illustrated by different colors (see legend). Significant differences (Tukey-Kramer, p < 0.05) are indicated by no common subscript letter. The number of animals can be seen below the boxes of the boxplot (e.g., n12 indicates 12 animals).

The interaction between genotype and age had a significant effect on keel bone ossification (F(6, 88) = 40.88, p < 0.001) (**Figure 7**). In all four genotypes, the ossification significantly proceeded between the 16^th^ and 24^th^ woa (p < 0.001) and between the 24^th^ and 33^rd^ woa (RHO, AL, and LB: p < 0.001, LSA: p = 0.004). Thereafter, the keel bone was fully ossified in all animals of all genotypes. In the 16^th^ woa, keel bone ossification was significantly further progressed in LSA than in RHO (p < 0.001) and showed a statistical trend of being further progressed than in AL (p = 0.059) with LB in between (p > 0.149). In the 24^th^ woa, ossification was significantly further progressed in AL, LB, and LSA than in RHO (RHO-AL and RHO-LSA: p < 0.001, RHO-LB: p = 0.002). In the 33^rd^ woa, there was no significant or significance approaching difference between the genotypes (p > 0.838).

**Figure 7:**
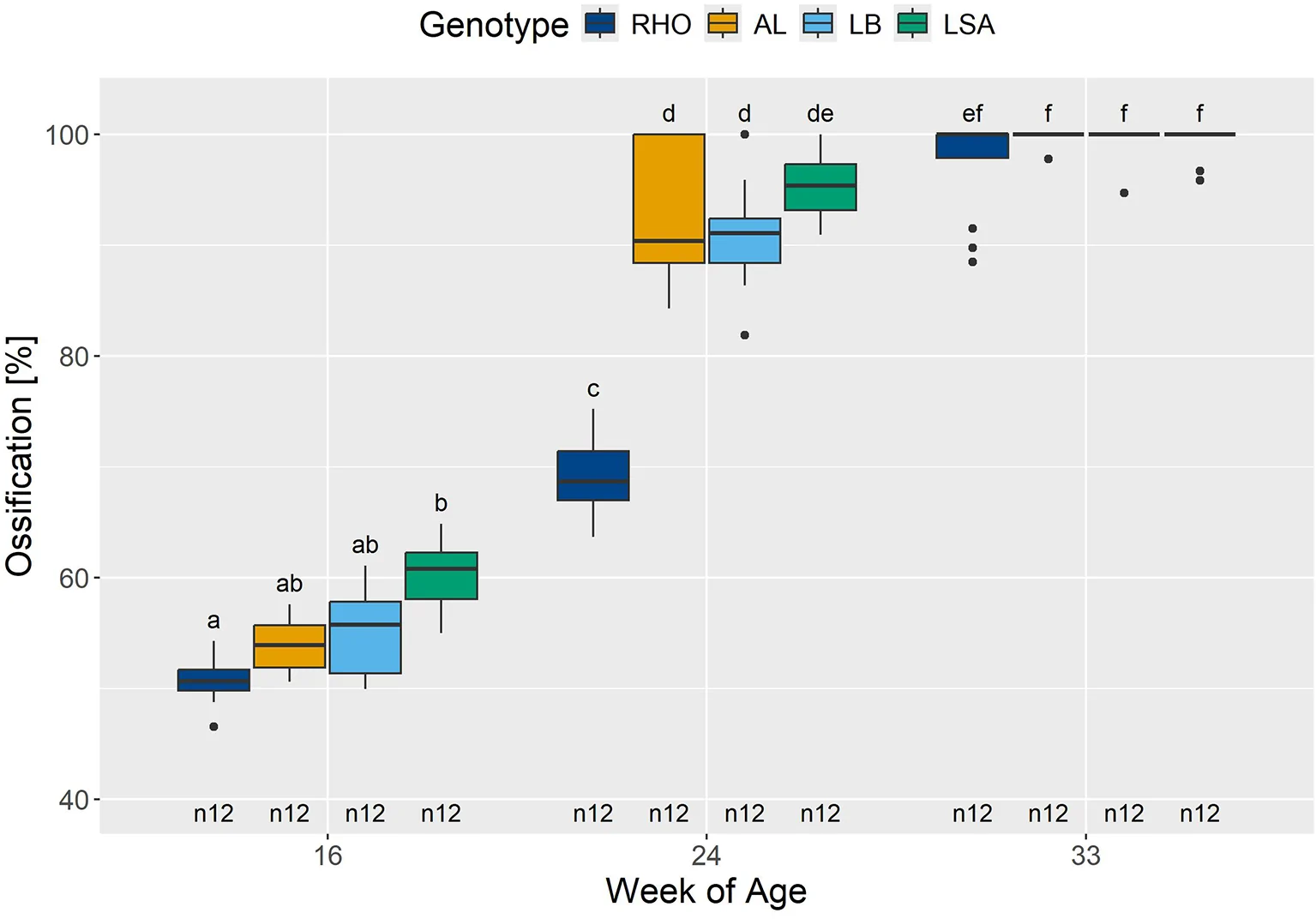
Keel bone ossification in relation to age and genotype. The x-axis represents age and the y-axis shows ossification in %. Different genotypes are illustrated by different colors (see legend). Significant differences (Tukey-Kramer, p < 0.05) are indicated by no common subscript letter. The number of animals can be seen below the boxes of the boxplot (e.g., n12 indicates 12 animals). The figure shows ossification until the 33^rd^ woa. Thereafter, keel bone ossification was completed in all animals.

### 3.5 Radiographic density and pectoral muscle thickness

Both radiographic density and pectoral muscle thickness were significantly influenced by the interaction between genotype and age (F(15, 207) = 11.33, p < 0.001 and F(12, 163) = 25.02, p < 0.001, respectively) (**Figures 8**, **9**). In all four genotypes, radiographic density increased between the 16^th^ and 47^th^ woa, but with different trajectories: RHO showed a significant increase between the 16^th^ and 33^rd^ woa (p < 0.001) and subsequently a further significant increase until the 47^th^ woa (p < 0.001). In AL, radiographic density tended to increase between the 16^th^ and 24^th^ woa (p = 0.057) and significantly increased between the 24^th^ and 33^rd^ woa (p > 0.001). In LSA, there were significant increases in radiographic density between the 16^th^ and 24^th^ as well as between the 24^th^ and 33^rd^ woa (p = 0.001 and p = 0.007, respectively). LB did not show significant changes in radiographic density between the 16^th^ and 33^rd^ woa (p > 0.908), but a significant increase between the 33^rd^ and 47^th^ woa (p < 0.001). Consequently, in the 33^rd^ woa, LB had significantly lower keel bone radiographic density than the other three genotypes (RHO-LB: p < 0.001, AL-LB: p = 0.017, LB-LSA: p = 0.022). From the 47^th^ woa onward, radiographic density was significantly higher in RHO than in the other three genotypes (47^th^ and 52^nd^ woa: p < 0.001, 72^nd^ woa: RHO-AL: p < 0.001, RHO-LB: p = 0.003, RHO-LSA: p = 0.002). Between the 47^th^ and 72^nd^ woa, keel bone radiographic density significantly decreased in RHO and AL (p = 0.019 and p = 0.002, respectively).

**Figure 8:**
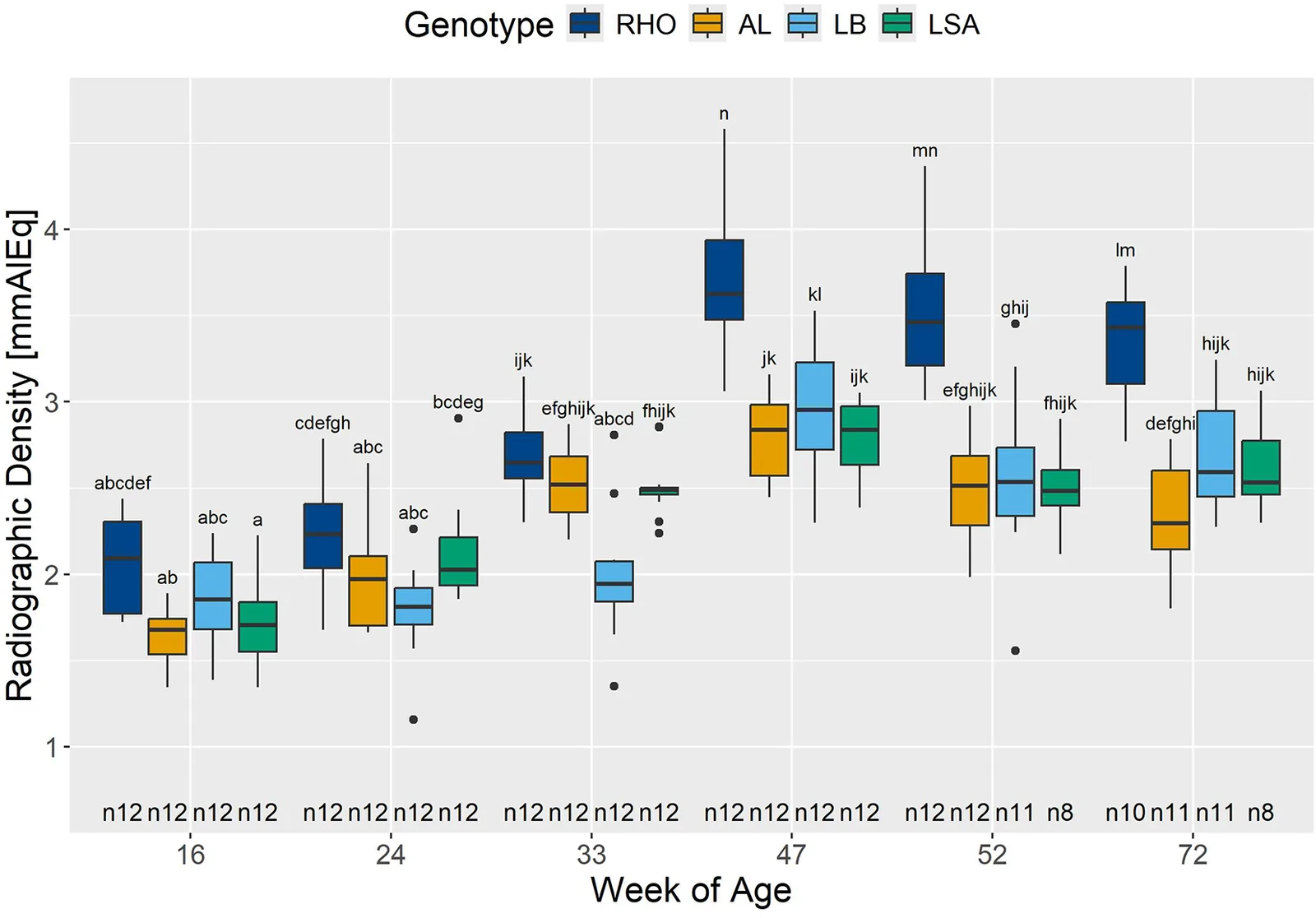
Radiographic density in relation to age and genotype. The x-axis represents age and the y-axis shows radiographic density in mm aluminum equivalent (mmAlEq). Different genotypes are illustrated by different colors (see legend). Significant differences (Tukey-Kramer, p < 0.05) are indicated by no common subscript letter. The number of animals can be seen below the boxes of the boxplot (e.g., n12 indicates 12 animals).

**Figure 9:**
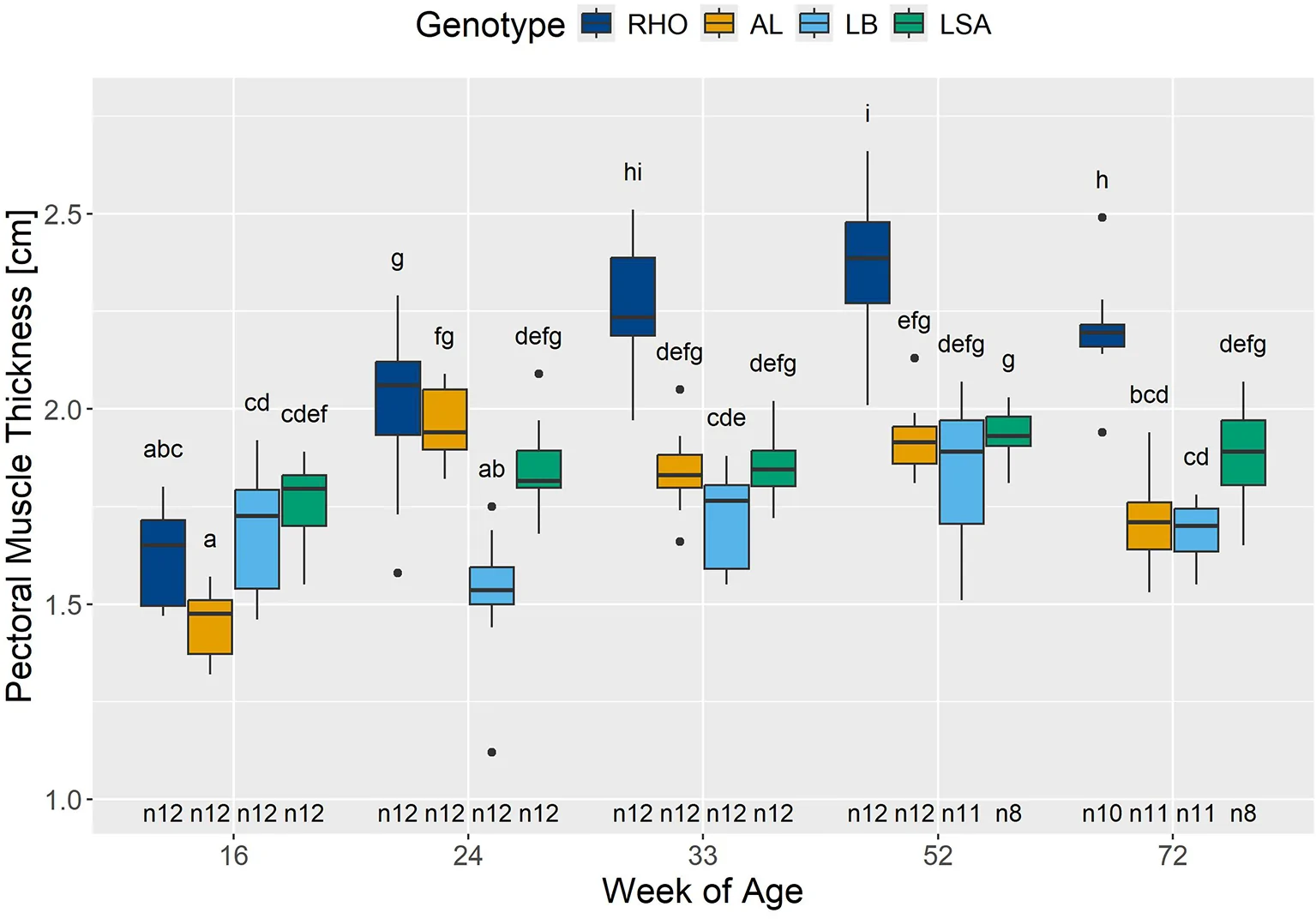
Pectoral muscle thickness in relation to age and genotype. The x-axis represents age and the y-axis shows pectoral muscle thickness in cm. Different genotypes are illustrated by different colors (see legend). Significant differences (Tukey-Kramer, p < 0.05) are indicated by no common subscript letter. The number of animals can be seen below the boxes of the boxplot (e.g., n12 indicates 12 animals).

In RHO, pectoral muscle thickness significantly increased between the 16^th^ and 24^th^ and between the 24^th^ and 33^rd^ woa (p < 0.001) and decreased between the 52^nd^ and 72^nd^ woa (p = 0.007). AL showed a significant increase in pectoral muscle thickness between the 16^th^ and 24^th^ woa and a significant decrease between the 24^th^ and 72^nd^ woa (p < 0.001). LB had significantly lower pectoral muscle thickness in the 24^th^ woa than at all other examinations (16^th^-24^th^ woa: p = 0.013, 24^th^-33^rd^ and 24^th^-52^nd^ woa: p < 0.001, 24^th^-72^nd^ woa: p = 0.034) and tended to have lower pectoral muscle thickness in the 72^nd^ woa than in the 52^nd^ woa (p = 0.085). LSA had significantly thicker pectoral muscles in the 52^nd^ woa than in the 16^th^ woa. In the 16^th^ woa, pectoral muscle thickness was lower in AL than in LB and LSA (p = 0.006 and p < 0.001, respectively). In the 24^th^ woa, LB had significantly lower pectoral muscle thickness than all other genotypes (p < 0.001), and from the 33^rd^ woa onward, RHO had significantly thicker pectoral muscles than the other three genotypes (33^rd^ and 52^nd^ woa: p < 0.001, 72^nd^ woa: RHO-AL and RHO-LB: p < 0.001, RHO-LSA: p = 0.001).

### 3.6 Plasma level of reproductive hormones

Both 17β-estradiol and FSH were significantly influenced by the interaction between age and genotype (F(12,146) = 5.62, p < 0.001 and F(12,141) = 9.42, p < 0.001, respectively) (**Figures 10**, **11**). In RHO, 17β-estradiol significantly increased between the 16^th^ and 24^th^ woa (p < 0.001) and between the 24^th^ and 33^rd^ woa (p < 0.001) and then leveled off (p > 0.645). AL, LB, and LSA showed a significant increase in 17β-estradiol from the 16^th^ to the 24^th^ woa (p < 0.001). While AL and LSA did not show significant changes thereafter (p > 0.164 and p > 0.308, respectively), LB had significantly higher 17β-estradiol plasma levels in the 52^nd^ than in the 33^rd^ woa (p = 0.040) and, furthermore, showed a tendency of having higher 17β-estradiol levels in the 72^nd^ than in the 33^rd^ woa (p = 0.074). In the 16^th^ woa, there was no significant difference between the genotypes (p > 0.485). In the 24^th^ woa, LB and LSA had significantly higher plasma 17β-estradiol levels than RHO (p < 0.001). From the 33^rd^ woa onward, there were no significant differences between the genotypes (p > 0.147).

**Figure 10:**
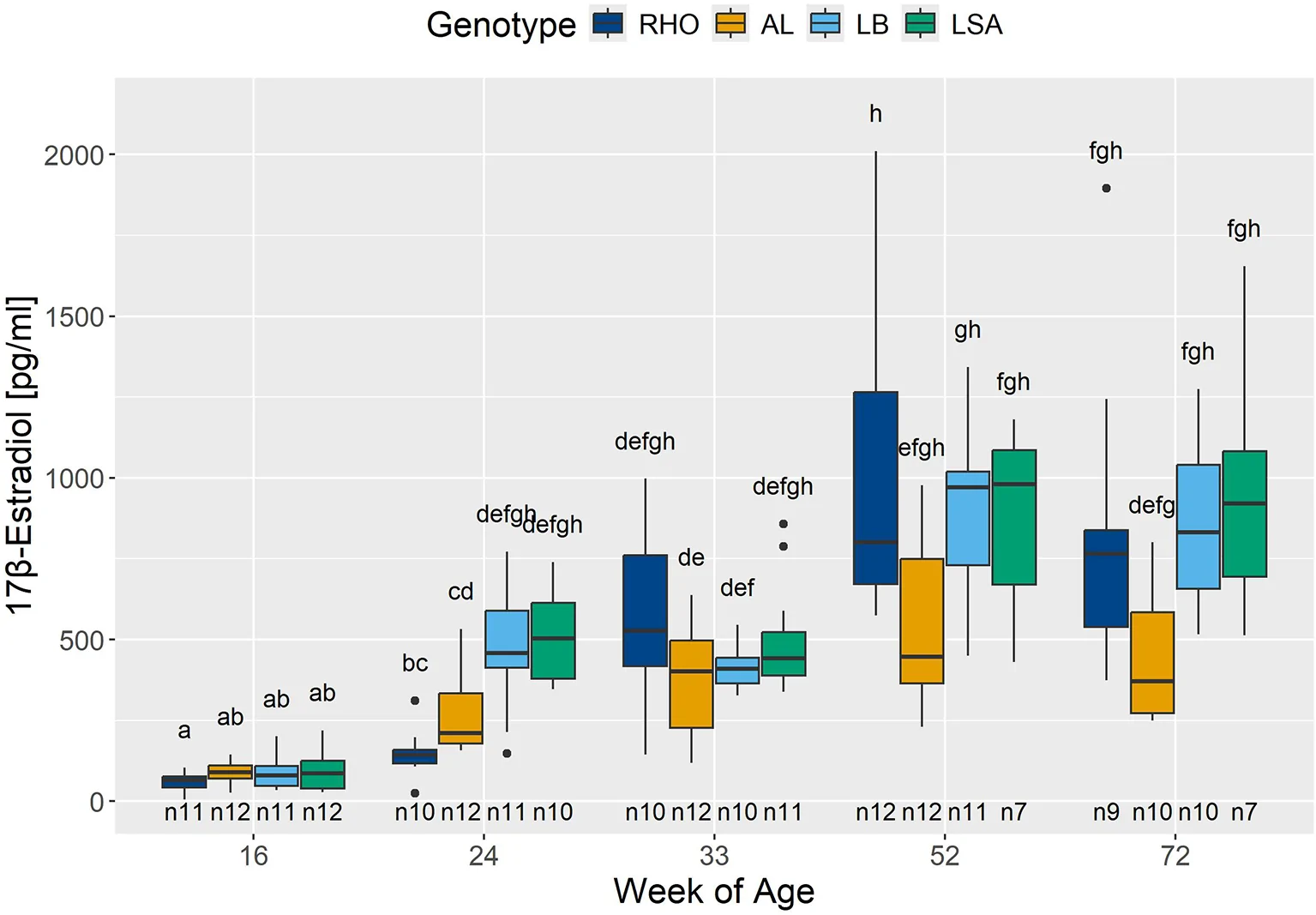
Plasma 17β-estradiol in relation to age and genotype. The x-axis represents age and the y-axis shows plasma concentration of 17β-estradiol in pg/ml. Different genotypes are illustrated by different colors (see legend). Significant differences (Tukey-Kramer, p < 0.05) are indicated by no common subscript letter. The number of animals can be seen below the boxes of the boxplot (e.g., n12 indicates 12 animals).

**Figure 11:**
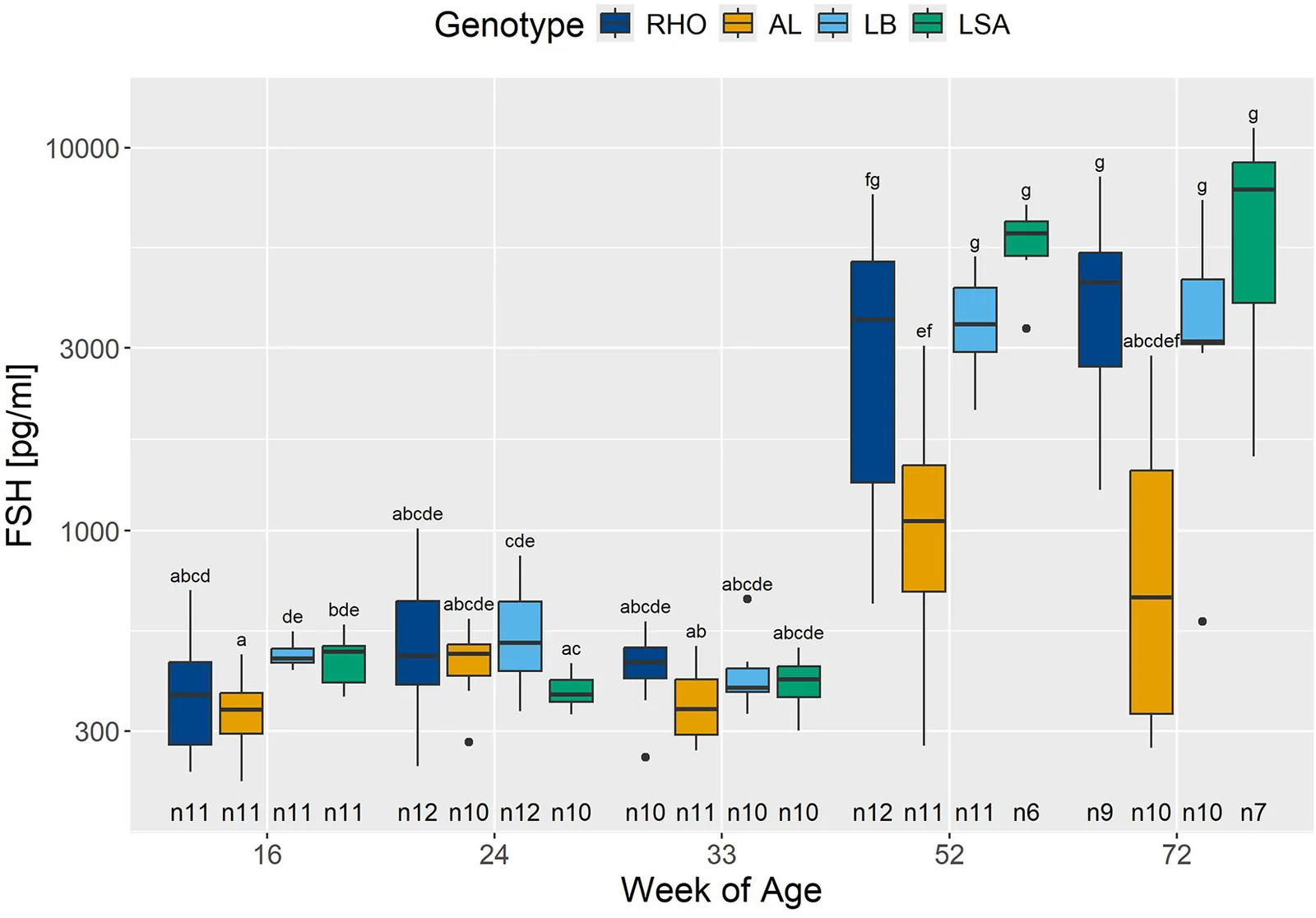
Plasma FSH in relation to age and genotype. The x-axis represents age and the y-axis (log-scaled) shows plasma concentration of FSH in pg/ml. Different genotypes are illustrated by different colors (see legend). Significant differences (Tukey-Kramer, p < 0.05) are indicated by no common subscript letter. The number of animals can be seen below the boxes of the boxplot (e.g., n12 indicates 12 animals).

In the 16th woa, plasma FSH was significantly higher in LB and LSA than in AL (AL-LB: p = 0.002, AL-LSA: p = 0.024) and in the 24th woa, LB tended to have higher FSH plasma concentrations than LSA (p = 0.091). In LSA, FSH levels were significantly lower in the 24^th^ than in the 16^th^ woa (p = 0.015). Between the 33^rd^ and 52^nd^ woa, all genotypes showed a significant increase in plasma FSH (RHO, LB, and LSA: p < 0.001, AL: p = 0.006). Consequently, in RHO, LB, and LSA, FSH was significantly higher in the 52^nd^ and 72^nd^ woa than before (p < 0.001), and in AL, plasma FSH levels were significantly higher in the 52^nd^ than in the 16^th^ and 33^rd^ woa (p = 0.003 and p = 0.006, respectively). In the 52^nd^ woa, AL had significantly lower FSH levels than LB and LSA (p < 0.001). In the 72^nd^ woa, AL showed significantly lower FSH levels than all other genotypes (AL-RHO: p = 0.002, AL-LB: p = 0.016, AL-LSA: p < 0.001).

### 3.7 Total calcium, ionized calcium and phosphate plasma levels

Total calcium was affected by both the interaction between follicle status and genotype (F(3, 165) = 19.41, p < 0.001) (**Figure 12**) and the interaction between age and genotype F(12, 162) = 48.25, p < 0.001) (**Table 4**). In all four genotypes, total calcium was significantly higher in animals *in-lay* (p < 0.001). Within the *non-laying* animals, LB had lower total calcium levels than RHO and AL (p = 0.001 and p = 0.012, respectively). Within the animals *in-lay*, LSA had significantly higher total calcium plasma levels than all other genotypes (RHO and AL: p < 0.001, LB: p = 0.023) and LB had significantly higher levels than AL (p = 0.032) and tended to have higher levels than RHO (p = 0.064).

**Figure 12:**
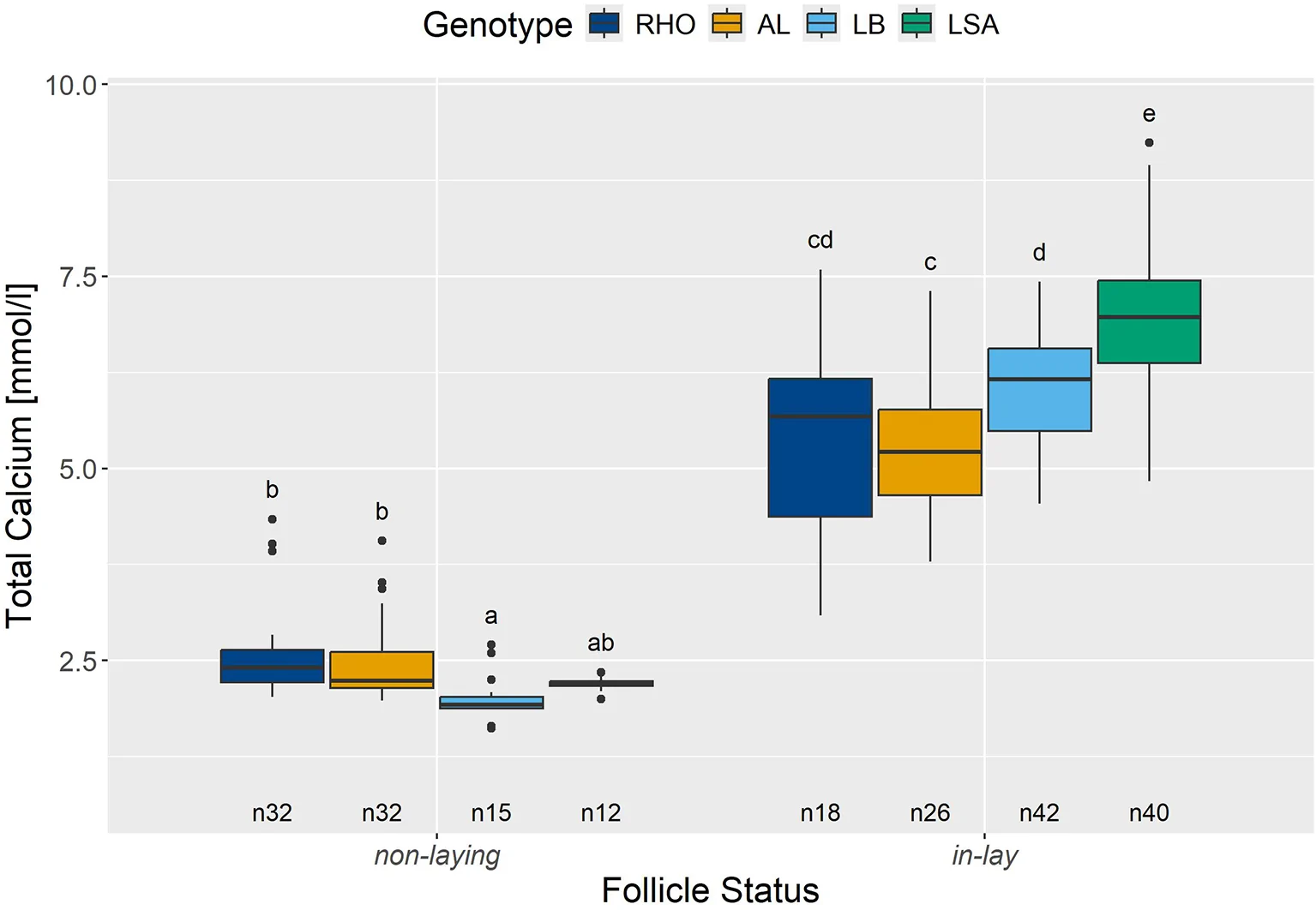
Plasma total calcium in relation to genotype and follicle status. The x-axis represents age and the y-axis shows plasma concentration of total calcium in mmol/l. Different genotypes are illustrated by different colors (see legend). Significant differences (Tukey-Kramer, p < 0.05) are indicated by no common subscript letter. The number of animals can be seen below the boxes of the boxplot (e.g., n32 indicates 32 animals).

**Table 4:** Least squares means and standard errors for plasma total calcium, blood ionized calcium, and plasma phosphate in relation to genotype and age.

Genotype x woa	Total Calcium [mmol/l]	Ionized Calcium [mmol/l]	Phosphate [mmol/l]
RHO x 16	2.21 ± 0.03 ^bc^	1.22 ± 0.04 ^abc^	2.44 ± 0.07 ^h^
RHO x 24	2.55 ± 0.05 ^d^	1.41 ± 0.04 ^cdefgh^	1.23 ± 0.06 ^b^
RHO x 33	3.65 ± 0.4 ^cdef^	1.39 ± 0.04 ^cdefgh^	1.63 ± 0.15 ^bcdefg^
RHO x 52	5.45 ± 0.31 ^fghi^	1.23 ± 0.04 ^abcd^	1.87 ± 0.11 ^efg^
RHO x 72	5.19 ± 0.35 ^efgh^	1.38 ± 0.04 ^bcdefgh^	1.66 ± 0.11 ^cdefg^
AL x 16	2.12 ± 0.02 ^b^	1.16 ± 0.04 ^ab^	2.1 ± 0.05 ^g^
AL x 24	2.94 ± 0.15 ^d^	1.54 ± 0.04 ^gh^	0.74 ± 0.06 ^a^
AL x 33	3.6 ± 0.36 ^de^	1.42 ± 0.04 ^cdefgh^	1.11 ± 0.11 ^abc^
AL x 52	5.94 ± 0.24 ^hijk^	1.24 ± 0.04 ^abcde^	1.72 ± 0.11 ^defg^
AL x 72	3.99 ± 0.45 ^defg^	1.39 ± 0.04 ^cdefgh^	1.14 ± 0.08 ^b^
LB x 16	1.88 ± 0.04 ^a^	1.33 ± 0.04 ^abcdef^	1.9 ± 0.06 ^efg^
LB x 24	5.72 ± 0.22 ^ghij^	1.43 ± 0.04 ^defgh^	1.24 ± 0.06 ^bcd^
LB x 33	4.92 ± 0.44 ^efgh^	1.51 ± 0.04 ^fgh^	1.46 ± 0.14 ^bcde^
LB x 52	6.4 ± 0.17 ^hijk^	1.44 ± 0.04 ^defgh^	1.85 ± 0.07 ^efg^
LB x 72	6.56 ± 0.17 ^hijk^	1.46 ± 0.04 ^fgh^	1.99 ± 0.09 ^efg^
LSA x 16	2.19 ± 0.03 ^bc^	1.13 ± 0.04 ^a^	1.78 ± 0.06 ^ef^
LSA x 24	7.02 ± 0.27 ^jk^	1.47 ± 0.04 ^fgh^	1.56 ± 0.09 ^bcde^
LSA x 33	6.57 ± 0.36 ^hijk^	1.44 ± 0.04 ^efgh^	1.78 ± 0.12 ^efg^
LSA x 52	6.89 ± 0.25 ^ijk^	1.33 ± 0.05 ^abcdefg^	1.84 ± 0.1 ^efg^
LSA x 72	7.52 ± 0.41 ^k^	1.59 ± 0.05 ^h^	2.4 ± 0.18 ^fgh^

Within a column, significant differences (Tukey-Kramer < 0.05) are indicated by no common subscript letters.

In all genotypes, plasma total calcium increased between the 16^th^ and 24^th^ woa (p < 0.001). While plasma total calcium did not change significantly in the other genotypes thereafter, AL showed a significant decrease in plasma total calcium between the 52^nd^ and 72^nd^ woa. Moreover, LB showed a tendency for having higher calcium levels in the 72^nd^ woa than in the 33^rd^ woa (p = 0.071). In the 16^th^ woa, LB had significantly lower plasma calcium levels than the other genotypes (p < 0.001). In the 24^th^ woa, LSA showed a tendency of having higher total calcium levels than LB (p = 0.053) and both had significantly higher calcium levels than the other two genotypes, RHO and AL (p < 0.001). In the 33^rd^ woa, LSA had significantly higher plasma calcium levels than RHO and AL (p < 0.001) with LB in between (p > 0.337). In the 52^nd^ woa, RHO showed a tendency of having lower calcium levels than LSA (p = 0.072), and in the 72^nd^ woa, LSA had significantly higher plasma calcium levels than RHO and AL (RHO: p = 0.011, AL: p < 0.001), while LB had significantly higher calcium levels than AL (p < 0.001) and tended to have higher levels than RHO (p = 0.094).

Ionized calcium and phosphate were influenced by the interaction between genotype and age, too (F(12, 163) = 3.13, p < 0.001 and F(12, 162) = 19.06, p < 0.001, respectively) (**Table 4**). In RHO and LB, there were no significant changes in ionized calcium with age, although in RHO it tended to be higher in the 24^th^ woa than in the 16^th^ woa (p = 0.070). AL and LSA had significantly higher ionized calcium levels in the 24^th^, 33^rd^, and 72^nd^ woa than in the 16^th^ woa (AL: 24^th^ woa: p < 0.001, 33^rd^ woa: p = 0.001, 72^nd^ woa: p = 0.010, LSA: p < 0.001). Furthermore, LSA had significantly higher ionized calcium levels in the 72^nd^ woa than in the 52^nd^ woa (p = 0.025). There were no significant differences between the genotypes at any time point. However, LB tended to have higher ionized calcium levels than LSA in the 16^th^ woa (p = 0.090) and RHO and AL tended to have lower ionized calcium levels than LB in the 52^nd^ woa (p = 0.059 and p = 0.073, respectively).

Phosphate levels in RHO were significantly higher in the 16^th^ woa than at any other age (p < 0.001) and significantly lower in the 24^th^ woa than in the 52^nd^ and 72^nd^ woa (p < 0.001 and p = 0.019, respectively) (**Table 4**). In AL, plasma phosphate was significantly higher in the 16^th^ woa than in the 24^th^, 33^rd^, and 72^nd^ woa (p < 0.001) and tended to be higher in the 16^th^ woa than in the 52^nd^ woa (p = 0.098). Furthermore, phosphate levels in AL were significantly higher in the 52^nd^ and 72^nd^ woa than in the 24^th^ week of age (p < 0.001 and p = 0.001, respectively). LB had significantly higher phosphate levels in the 16^th^, 52^nd^, and 72^nd^ woa than in the 24^th^ woa (p < 0.001) and showed a tendency of having lower phosphate levels in the 33^rd^ than in the 72^nd^ woa (p = 0.090). In LSA, phosphate levels in the 72^nd^ woa were significantly higher than in the 24^th^ woa (p = 0.004) and tended to be higher than in the 16^th^ woa (p = 0.082). In the 16^th^ woa, RHO had significantly higher phosphate levels than the other three genotypes (RHO-AL: p = 0.035, RHO-LB and RHO-LSA: p < 0.001) and AL had significantly higher phosphate levels than LSA (p = 0.016). In the 24^th^ woa, AL had significantly lower plasma phosphate than the other three genotypes (p < 0.001) and in the 33^rd^ woa, plasma phosphate in AL was significantly lower than in LSA (p = 0.020) with RHO and LB in between (p > 0.429). In the 52^nd^ woa, there were no significant differences between the genotypes (p > 0.999). In the 72^nd^ woa, AL had significantly lower plasma phosphate than all other genotypes (RHO-AL: p = 0.040, AL-LB and AL-LSA: p < 0.001) and RHO tended to have lower plasma phosphate than LSA (p = 0.081).

## 4 Discussion

In the current study, the selection for economic performance was clearly displayed in the performance data of the hybrid layers, which showed a markedly higher laying performance compared to the two traditional breeds. In addition, clear differences in keel bone damage were seen between the genotypes. As hypothesized, LB and LSA showed significantly more keel bone fractures than the two traditional breeds, while the genotypes did not significantly differ in terms of deviation severity, as assessed through the measurement of POD. Moreover, differences in body weight, keel bone parameters, and pectoral muscle thickness indicate that the genotypes differ in body composition and development. In this regard, especially RHO stood out due to their presumably larger body size and slower development. While plasma total calcium and 17β-estradiol clearly increased when the hens entered the laying period, results on ionized calcium, phosphate, and FSH indicate that these were influenced by other factors.

### 4.1 Production parameters

#### 4.1.1 Body weight

Body weight should be kept in mind when comparing different genotypes in terms of morphological parameters, such as keel bone length, radiographic density, and pectoral muscle thickness. For example, body weight can correlate with bone density and size (Habig et al., 2021a; Kolakshyapati et al., 2019).

Across all genotypes, mean body weight in the 52^nd^ and 72^nd^ woa was at the upper end or slightly exceeded the range given by the BDRG and the breeding company (RHO: 2.4 to 3 kg, AL: 1.7 to 2.2 kg, LB and LSA: 2 kg, (Bund Deutscher Rassegeflügelzüchter e.V, n.d.; LOHMANN BREEDERS GmbH, 2021a, LOHMANN BREEDERS GmbH, 2021b)). As expected, based on this information, RHO were significantly heavier than the other genotypes. The initial increase of body weight could be caused by overall body growth but also by an increase in organ mass when the animals enter lay (Kwakkel et al., 1995). Therefore, the later onset of lay in RHO may explain at least part of the weight gain observed after the 24^th^ woa compared to the other genotypes. AL weighing significantly less than the two hybrid layers in the 16^th^ and 24^th^ woa despite reaching comparable adult body weight could suggest differences in body development, i.e., slower or later growth in the less intensively selected AL. Possible explanations for the increase in body weight in LB between the 33^rd^ and 52^nd^ woa are the relocation, the associated decrease in pen size, and the change from pelleted to meal feed. However, usually, weight gain and feed intake is higher with pelleted feed (Wan et al., 2021).

#### 4.1.2 Onset of lay

The first egg was laid distinctly earlier in LB and LSA (18^th^ and 19^th^ woa, respectively) than in AL (23^rd^ woa) and RHO (30^th^ woa). Although the housing conditions did not resemble the usual conventional housing conditions, the onset of lay in LB and LSA aligns with the information from the breeding company (LOHMANN BREEDERS GmbH, 2021a, LOHMANN BREEDERS GmbH, 2021b). In the past, early onset of lay has been an important selection criterion to improve performance and economic efficiency, as it strongly influences the number of eggs a hen lays within a specific time period (Brown, 1915; Thiruvenkadan et al., 2010).

Based on the results on body weight and first eggs, RHO stood out by being distinctly heavier and showing slower growth and later sexual maturation than the other breeds. This should be kept in mind, as it affects the comparability of morphological traits (e.g., keel bone length, radiographic density, pectoral muscle mass) and blood parameters during the early phase of the study.

#### 4.1.3 Laying performance

The average number of eggs per hen within the first laying year in LB (318 eggs/year) and LSA (327 eggs/year) matches the performance data from the breeding company (LOHMANN BREEDERS GmbH, 2021a, LOHMANN BREEDERS GmbH, 2021b). The laying performance of RHO (165 eggs/year) and AL (142 eggs/year) was distinctly lower and did not reach the expectations based on the information of the BDRG (Bund Deutscher Rassegeflügelzüchter e.V.). It should be considered that this information might not be accurate, as there is no systematic performance testing of non-commercial breeds. A further possible explanation for this discrepancy is that the chickens in this study were kept without artificial lighting program but under natural lighting conditions. This is in agreement with the information from the literature indicating that the influence of the photoperiod on laying performance is higher on traditional breeds than on modern chicken lines and hybrids (Lewis et al., 1997; Morris et al., 1995; Okumura et al., 1988). In accordance with that, laying performance strongly fluctuated throughout the year in RHO and AL but not in the two hybrids. RHO and AL exhibited their peak laying performance at different time points. AL and RHO reaching peak performance at different times of the year could indicate genotype-specific differences in the response to changes in photoperiod.

### 4.2 Prevalence and severity of keel bone damage

In this study, the two hybrid layers LB and LSA showed significantly more keel bone fractures than RHO and AL. This aligns with the findings of other authors who found fewer fractures in low-performing genotypes and traditional breeds than are usually found in hybrid layers (Hildebrand et al., 2026; Kittelsen et al., 2020, Kittelsen et al., 2021; Pulcini et al., 2023). Nevertheless, the overall prevalence of keel bone fractures in RHO (0%) and AL (9.1%) was at the lower end of the range reported for traditional breeds by Jung et al. (2024) who found distinctly higher prevalence rates in some traditional breeds. However, it should be noted that the authors did not use radiography but palpation and that the animals were kept under different housing conditions, which could have influenced the risk for keel bone fractures. A contributing factor for the low number of keel bone fractures in the two traditional breeds in our study could have been that the accessible heights (e.g., of perches) being relatively low compared to other housing systems. Nevertheless, LB and LSA showed high prevalence of keel bone fractures (90% and 87.5%, respectively) indicating that the potential beneficial effect of the housing conditions did not suffice to reduce fracture prevalence in the hybrid layers. This supports the hypothesis that not high impact events but insufficient bone stability are the main cause for the high prevalence of keel bone fractures observed in commercial layers.

There was an increase in fracture prevalence between the 33^rd^ and 47^th^ woa, when the animals were relocated. However, in the 47^th^ woa, only four out of 15 fractured keel bones showed visible fracture gaps. Since fracture healing takes about six weeks in birds (Dascălu et al., 2013), it is unlikely that the other fractures occurred at or after the relocation. In addition, an increasing fracture prevalence at that age has also been reported by other authors (Baur et al., 2020; Emmert et al., 2024; Petrik et al., 2015). Thus, although the relocation may have increased the risk for new fractures, it seems not to be the main cause of increase in fracture prevalence between these time points.

The prevalence of keel bone deviations varied in relation to age and genotype. In the 72^nd^ woa, the prevalence of keel bone deviations was highest in LSA followed by LB and RHO. However, overall, the POD of the detected deviations was relatively low (max. 2.04%) compared to other studies (Eusemann et al. (2018): cage housed: max. 36.23%, floor housed: max. 9.52%). Nevertheless, severity in this study was comparable to the previous study, where animals were housed under similar conditions (Hildebrand et al. (2026): max. 5.37%). The high space allowance and early access to outdoor enclosures in these studies may have contributed to preventing further aggravation of keel bone deviations. Besides the beneficial effect of exercise on bone stability, the exposure to natural sunlight may have supported bone health by promoting the synthesis of Vitamin D, which is the precursor of calcitriol – a hormone that enhances calcium uptake (as reviewed by Sinclair-Black et al., 2023). However, this has not been further investigated in this study.

In some cases, animals showed a deviation at one but not at the subsequent radiographic examination. While this could indicate recovery from minor keel bone deviations (Warren, 1937), it also could demonstrate poor repeatability for the detection of minor deviations using X-ray imaging (Tracy et al., 2019). In latero-lateral X-ray images, only sagittal deviations are clearly visible. Transversal deviations, however, can stay undetected or be underestimated using this method. Overall, the biological relevance of keel bone deviations observed in this study remains unclear. Future research should investigate the impact of keel bone deviations with different severities on the animal.

### 4.3 Keel bone length and ossification

The initial increase of keel bone length supports the hypothesis that at least some of the weight gain observed was due to body growth. Changes in keel bone length later in the laying period have previously been reported and it has been proposed that shortening occurs as part of fracture healing (Baur et al., 2020). However, this is a rather unlikely explanation for the decrease of keel bone length in RHO as no fractures were found in this genotype during the study. A different explanation for the decrease in the measured length is that the keel bones became more curved with age, which would shorten the distance between the cranial and caudal tip of the keel bone. Throughout the study, RHO had longer keel bones than the other genotypes, comparable to their larger body size compared to the other genotypes. However, while not significantly differing in body weight, AL had significantly longer keel bones than LB in the 24^th^ woa and tended to have longer keel bones in the 33^rd^ woa. This aligns with the findings of Fawcett et al. (2020) that white layers had longer keel bones than brown layers and indicates differences in body structure between the genotypes.

Regarding keel bone ossification, there were no significant differences between AL, LB, and LSA. This confirms the findings from a previous study that earlier sexual maturity does not always correspond with earlier completion of keel bone ossification (Hildebrand et al., 2026). Since LB and LSA lay their first egg five and four weeks earlier than AL, respectively, these findings suggest that keel bone ossification is less progressed in the two hybrid layers than in AL at onset of lay. However, since no investigation took place at onset of lay, the exact extent of keel bone ossification at this time point remains unclear. Further investigations should take place to explore the exact ossification timeline in hybrid layers in relation to onset of lay. Keel bone ossification in RHO progressed slower than in the other genotypes. Taken together with the findings from other studies (Buckner et al., 1948; Hildebrand et al., 2026), this could indicate that in large, slow-developing genotypes, ossification is completed later.

### 4.4 Radiographic density and pectoral muscle thickness

When interpreting radiographic density, body weight and musculature should be taken into account. On the one hand, the tissue surrounding the bone contributes to the gray value measured as radiographic density (Allen and Krohn, 2013). On the other hand, body weight and muscle mass are positively associated with bone density (Habig et al., 2021a; Qin and Jiao, 2022). The progressive increase in radiographic density between the 16^th^ and 47^th^ woa in RHO, AL, and LSA is similar to the patterns in other genotypes as observed in previous studies (Eusemann et al., 2020; Hildebrand et al., 2026). In LB, however, the keel bone radiographic density increased later, between the 33^rd^ and 47^th^ woa. Although this could mean a late increase in bone mineralization, it could also be linked to the animals’ body condition.

In contrast to the other genotypes, pectoral muscle thickness significantly decreased in LB between the 16^th^ and 24^th^ woa. This could indicate that body condition in LB decreased, even if the animals gained weight. Consequently, LB had significantly thinner pectoral muscles than the other genotypes in the 24^th^ woa. Comparable to our findings, Fawcett et al. (2020) found significantly lighter pectoral muscles in LB compared to white layers, but earlier, at 16 woa.

The longer increase in pectoral muscle thickness and greater pectoral muscle thickness from the 33^rd^ woa onward in RHO again illustrate the slower development and larger body size compared to the other genotypes. Between the 52^nd^ and 72^nd^ woa, decreases in pectoral muscle thickness and radiographic density were observed in RHO and AL. In an experiment conducted by Garant et al. (2023), white hens with immobilized wings showed a significant decrease in pectoral thickness over a period of 5 weeks. Likewise, it is possible that, in the present study, the decrease in exercise following the relocation to pens with a smaller run area led to a decrease in pectoral muscle thickness. Although the observer for the measurement of pectoral muscle thickness did not change between the data collections, observer related variations cannot be excluded.

### 4.5 Plasma concentration of reproductive hormones

In accordance with previous studies, plasma 17β-estradiol increased around onset of lay in all genotypes (Habig et al., 2021b; Mehlhorn et al., 2022). The increase in plasma 17β-estradiol that was observed in RHO between the 16^th^ and 24^th^ woa before they entered lay could refer to rising estradiol levels during puberty, as reported in humans (Visser, 1973). While RHO, AL, and LSA did not show significant changes after their onset of lay, estradiol levels in LB significantly increased between the 33^rd^ and 52^nd^ woa. The results on body weight and radiographic density in this hybrid indicate an increase in body condition at this time. In humans, body fat and energy balance influence 17β-estradiol levels (Ziomkiewicz et al., 2008). Although the graph indicated a lower 17β-estradiol level in AL, no significant or significance approaching differences were detected between the genotypes from the 33^rd^ woa onward. It should be noted that, due to the relatively high intragroup variances and small animal numbers, the difference would have had to be relatively strong to be considered statistically significant. The high intragroup variances are in agreement with the findings of other authors who reported high fluctuations in plasma 17β-estradiol and large variations between individual birds (Leszczynski et al., 1985; Tanabe et al., 1981). As mentioned before, 17β-estradiol induces the formation of medullary bone that has been linked to the loss of structural bone (Squire et al., 2017; Whitehead, 2004; Wilson and Thorp, 1998). However, the potentially negative influence of 17β-estradiol appears to depend on laying activity. In Eusemann et al. (2020), non-laying hens with exogenous estrogen showed an even lower risk for keel bone fractures than non-laying hens without estrogen treatment. It is furthermore conceivable that not the concentration level, but the time and duration of elevated estradiol levels may be decisive for the impact on bone stability. It has been described that new structural bone is formed, when a hen goes out of lay (Whitehead and Fleming, 2000). Continuously high estradiol levels may prohibit this recovery of structural bone. In addition, elevated estradiol levels before complete keel bone ossification could have an impact on the newly formed bone tissue and, therefore, impair bone quality and stability in the caudal part of the keel bone.

Both hybrid layers having significantly higher FSH levels than AL in the 16^th^ week of age, and LSA showing lower plasma FSH in the 24^th^ than in the 16^th^ woa could be attributable to a peak in FSH plasma concentration at onset of sexual maturity, as observed by Hanlon et al. (2021). All four genotypes showed a significant increase in plasma FSH between the 33^rd^ and 52^nd^ woa, when the seasons shifted from winter to spring and the daily photoperiod increased from 8:20 to 12:52 hours a day. Increases in FSH at this time of the year have been reported from other avian species living under natural daylight conditions, e.g., geese and great tits (Silverin et al., 1997; Yang et al., 2017). Although the absolute values of FSH differ between the studies, the relative increase between short and long days is in agreement with our findings. At this point, it should be noted that FSH plasma concentrations measured in chickens strongly vary across different studies (Dunn et al., 2003; Onagbesan et al., 2006; Prastiya et al., 2022). This could be due to the usage of different assays to measure the hormone. We therefore suggest treating the absolute values with caution and focusing on group and age differences.

Seeing this strong effect of seasonal changes on FSH in all four genotypes was unanticipated. Since FSH is involved in the recruitment of follicles, a positive relationship between FSH levels during the laying period and laying intensity was expected. However, laying activity in modern hybrids is relatively independent from photoperiodic changes. Even though photoperiodic stimulation, i.e., changing from shorter to longer days, is known to bring the onset of lay up to 3 to 4 weeks forward (Lewis et al., 1997), it does not seem to have a great impact on laying performance during the laying period, as they do already exhibit good laying persistency over the course of constantly long days (Morris et al., 1995). This was also the case in our study. A possible explanation, why the increase in plasma FSH did not reflect in the laying intensity of the hybrid layers, is that they already approach the physiological maximum of one egg per day at lower FSH levels, so that higher FSH levels do not further enhance laying intensity. Regarding the differences between the genotypes, AL having lower FSH levels than the other genotypes in the 52^nd^ and 72^nd^ woa and lower laying intensity, could indeed indicate a positive relationship between plasma FSH and laying intensity. This aligns with the findings of Prastiya et al. (2022), who observed higher FSH levels in animals that laid eggs daily than hens with lower laying performance.

In summary, our results highlight the complexity of the regulation of FSH secretion and the relationship with laying activity. Due to the small animal numbers and the lack of individual data on laying activity, the interpretability is limited. Further studies should be conducted to investigate this aspect in detail.

### 4.6 Blood plasma minerals

Total plasma calcium was highly influenced by laying activity with hens *in-lay* showing significantly higher total plasma calcium levels than *non-laying* hens and plasma calcium significantly increasing around onset of lay in all genotypes. This is in line with several other studies reporting higher total calcium levels during the laying phase (Habig et al., 2021b; Hanlon et al., 2022a). Within the hens *in-lay*, LB had significantly higher total calcium levels than AL and LSA had significantly higher plasma calcium concentrations than all other genotypes. This trend of hybrid hens having higher plasma calcium levels was also observed when analyzed in relation to age. In our study, LB and LSA showed distinctly higher laying performance than the other two genotypes. The higher total calcium plasma levels may reflect increased calcium transport in the blood to meet the increased calcium demand for the formation of the eggshell compared to lower-performing genotypes. Higher total plasma calcium levels in high compared to low-performing genotypes were also observed in the previous study (Hildebrand et al., 2026).

LB showing significantly lower calcium levels than the other genotypes in the 16^th^ woa is comparable to findings by Habig et al. (2021b), who saw lower total calcium levels in a brown-laying line than in a white-laying line at 17 woa, and could indicate genotype dependent differences in calcium plasma concentration before onset of lay. Regarding *non-laying* LB showing significantly lower total plasma calcium levels than *non-laying* AL and RHO, it should be noted that there is a strong association between age and laying activity in the hybrid layers. Since they lay more or less continuously after onset of lay, the vast majority of *non-laying* LB and LSA hens were animals before onset of lay, i.e., in the 16^th^ woa. Notably, calcium plasma concentration decreased in AL between the 52^nd^ and 72^nd^ woa while staying stable in the other genotypes. This is in agreement with the observed decrease in laying activity around that time.

Although both ionized calcium and phosphate levels were highly variable, different patterns could be observed. Between the 16^th^ and 24^th^ woa, when the animals entered lay, ionized calcium significantly increased in AL and LSA and tended to increase in LB. This is in agreement with the findings of Habig et al. (2021b). At the same time, phosphate levels significantly decreased in LB, RHO, and AL. This aligns with the findings of Hildebrand et al. (2026); Wei et al. (2021), and Qasir et al. (2025), but not with Habig et al. (2021b), who saw an increase of plasma phosphate at onset of lay. Moreover, in some genotypes, phosphate levels tended to increase in the later laying period. In AL, phosphate levels were higher in the 52nd woa, i.e., close to when they reached peak performance, compared to the 24th, 33rd, and 72nd woa and both LB and LSA showed a significant increase in phosphate between the 24th and 72nd woa. This is in accordance with our previous findings in Hildebrand et al. (2026) on Lohmann Selected Leghorn and could indicate increased mobilization of bone substance (Habig et al., 2021b; Parsons and Combs, 1981). However, it should be taken into account that both ionized calcium and phosphate are influenced by several other factors, such as feed intake, time of the day, and measuring method (as discussed in Hildebrand et al., 2026).

### 4.7 Association of production and bone parameters with keel bone damage

So far, the etiology of keel bone fractures is not fully clear. Potential internal risk factors include laying performance and early onset of lay. Furthermore, associations have been made between keel bone shape and fractures. In the following, we discuss if and how our findings support the existing hypotheses.

Across different studies and with few exceptions, lower performing genotypes appear to experience fewer keel bone fractures than commercial hybrid layers (Jung et al., 2024; Kittelsen et al., 2020, Kittelsen et al., 2021; Pulcini et al., 2023; Thøfner et al., 2021; Wilkins et al., 2011). This was also the case in our study. Taken together, these findings clearly show strong differences between genotypes and indicate that the selection for egg production traits may have led to an increased fracture risk in the high-performing layers. However, a strong association between individual laying performance and bone strength was not observed within one layer line, when hens that paused laying were excluded from the analysis (Jansen et al., 2020). Thus, it is important to distinguish between selection for egg production traits and individual laying performance within a genotype. In this context, it should also be kept in mind that the difference in laying performance is much bigger between the genotypes than between individuals of the same layer hybrid. Furthermore, it has to be taken into account that the selection for egg production traits does not only target the egg number but also other traits (e.g., onset of sexual maturity, feed efficiency, egg quality) that possibly contribute to this outcome (Flock and Preisinger, 2007).

In our study, RHO and AL did not only lay less eggs in total, but showed, in contrast to the hybrid layers, more pronounced seasonal fluctuations in laying intensity. When a hen goes out of lay, medullary bone recedes and new structural bone is built (Whitehead and Fleming, 2000). Thus, regular seasonal laying pauses may play a role in maintaining bone stability at an adequate level and therefore reduce keel bone fractures.

Early onset of lay, before completion of keel bone ossification, has been stated as a potential risk factor for keel bone fractures by several authors (Gebhardt-Henrich and Fröhlich, 2015; Jung et al., 2019; Kittelsen et al., 2020; Thøfner et al., 2020). In our study, LB and LSA laid their first egg distinctly earlier than the other two genotypes. Although the comparability with RHO is limited, it can be assumed that keel bone ossification and mineralization did not proceed faster than in AL and were therefore less progressed at onset of lay in the laying hybrids. This may lead to the keel bone being more susceptible to potential harmful influences, such as the competitive calcium situation and high levels of estradiol, impair bone stability and increase the risk for keel bone fractures.

Finally, keel bone shape has recently been associated with the occurrence of keel bone fractures. As mentioned before, the observed shortening of the keel bones of RHO possibly indicates an increase in keel bone curvedness with age. The resulting concave area on the visceral face of the keel bone has been proposed as a possible selection criterion for keel bone health (Sallam et al., 2025). Associations between keel bone shape and fractures have further been made by Pulcini et al. (2023), who saw less fractures but simultaneously more S-shaped deviations in traditional Italian breeds compared to Lohmann Brown hens. Although keel bone shape has not been investigated, our results suggest that an association between keel bone shape or curvedness and keel bone fractures may also exist in the genotypes examined in this study.

Although the comparison of different genotypes with divergent features helps to identify potential risk factors and support existing hypotheses, a clear distinction between critical and confounding factors is difficult. We therefore support a more detailed investigation of the factors mentioned above.

## 5 Conclusion

In conclusion, this study provides insights into the longitudinal development of keel bone damage and the values of associated performance, blood, and bone parameters in two traditional breeds and two hybrid layers. Compared to the hybrid layers, the traditional breeds in this study showed fewer keel bone fractures, lower laying performance with seasonal fluctuation, and presumably further progressed keel bone mineralization and ossification at onset of lay. Despite the high divergence in laying performance, there were no clear differences in the plasma concentrations of FSH and 17β-estradiol during the laying period. Given the exploratory nature of this study, future research should address the endocrinology of different genotypes in more detail, including the influence of changes in light duration.

## Data Availability

The datasets presented in this study can be found in online repositories. The names of the repository/repositories and accession number(s) can be found below: 10.5281/zenodo.21997166.

## References

[B1] Adler C. A. B. Shynkaruk T. McPhee S. Buchynski K. Herr A. Herwig E. . (2024). Balancing act: Studying the effect of perch space allowance on welfare in Canadian laying strain pullets raised in floor pens with access to a single-tier perch system to 18 wk of age. Poult Sci. 103, 104457. doi: 10.1016/j.psj.2024.104457 39504835 PMC11570713

[B2] Allen M. R. Krohn K. (2013). “ Skeletal imaging,” in Basic and Applied Bone Biology. Eds. Burr D. B. Allen M. R. ( Academic Press, Amsterdam, Heidelberg), 93–113.

[B3] Armstrong E. A. Rufener C. Toscano M. J. Eastham J. E. Guy J. H. Sandilands V. . (2020). Keel bone fractures induce a depressive-like state in laying hens. Sci. Rep. 10, 3007. doi: 10.1038/s41598-020-59940-1 32080271 PMC7033198

[B4] Farm Animal Welfare in Great Britain: Past, Present and Future. (2009). Available online at: https://assets.publishing.service.gov.uk/media/5a7d89fe40f0b64fe6c24508/Farm_Animal_Welfare_in_Great_Britain_-_Past:Present_and_Future.pdf (Accessed September 8, 2026).

[B5] Baur S. Rufener C. Toscano M. J. Geissbühler U. (2020). Radiographic evaluation of keel bone damage in laying hens-morphologic and temporal observations in a longitudinal study. Front. Vet. Sci. 7, 129. doi: 10.3389/fvets.2020.00129 32226794 PMC7081720

[B6] Brown E. (1915). Time of hatching pullets in relation to period of laying and total egg-production. Illustrated Poultry Rec. 7, 95–100.

[B7] Buckner G. D. Insko W. M. Henry A. H. Wachs E. F. (1948). Rate of growth and calcification of the sternum of male and female New Hampshire chickens. Poult Sci. 27, 430–433. doi: 10.3382/ps.0270430

[B8] Bund Deutscher Rassegeflügelzüchter e.V Rassetafeln Huehner. Available online at: https://www.bdrg.de/media/docs/Rassetafeln_Huehner.pdf (Accessed September 8, 2026).

[B9] Casey-Trott T. Guerin M. T. Sandilands V. Torrey S. Widowski T. M. (2017). Rearing system affects prevalence of keel-bone damage in laying hens: a longitudinal study of four consecutive flocks. Poult Sci. 96, 2029–2039. doi: 10.3382/ps/pex026 28371938

[B10] Casey-Trott T. Heerkens J. L. T. Petrik M. Regmi P. Schrader L. Toscano M. J. . (2015). Methods for assessment of keel bone damage in poultry. Poult Sci. 94, 2339–2350. doi: 10.3382/ps/pev223 26287001

[B11] Dascălu R. Sabău M. Proteasa A. Schuszler L. Sala A. Şerb M. . (2013). Contributions to the treatment of traumatic orthopedic disorders in birds. Sci. Works Ser. C Vet. Med. 59, 77–84.

[B12] DePaoli E. Korver D. Bench C. (2024). The effect of laying hen strain on perching biomechanics and keel deviations. J. Appl. Poult Res. 33, 100494. doi: 10.1016/j.japr.2024.10049442574925

[B13] Dunn I. C. Lewis P. D. Wilson P. W. Sharp P. J. (2003). Acceleration of maturation of FSH and LH responses to photostimulation in prepubertal domestic hens by oestrogen. Reproduction 126, 217–225. doi: 10.1530/rep.0.1260217 12887278

[B14] Emmert B. J. Regmi P. Robison C. I. Kim W. K. Karcher D. M. (2024). Utilizing 3-dimensional models to assess keel bone damage in laying hens throughout the lay cycle. Poult Sci. 103, 103804. doi: 10.1016/j.psj.2024.103804 38776860 PMC11131070

[B15] Eusemann B. K. Baulain U. Schrader L. Thöne-Reineke C. Patt A. Petow S. (2018). Radiographic examination of keel bone damage in living laying hens of different strains kept in two housing systems. PloS One 13, 1–17. doi: 10.1371/journal.pone.0194974 29742164 PMC5942800

[B16] Eusemann B. K. Patt A. Schrader L. Weigend S. Thöne-Reineke C. Petow S. (2020). The role of egg production in the etiology of keel bone damage in laying hens. Front. Vet. Sci. 7. doi: 10.3389/fvets.2020.00081 32154276 PMC7047165

[B17] Farmer M. Roland D. A. Clark A. J. (1986). Influence of dietary calcium on bone calcium utilization. Poult Sci. 65, 337–344. doi: 10.3382/ps.0650337 3703779

[B18] Fawcett D. L. Casey-Trott T. M. Jensen L. Caston L. J. Widowski T. M. (2020). Strain differences and effects of different stocking densities during rearing on the musculoskeletal development of pullets. Poult Sci. 99, 4153–4161. doi: 10.1016/j.psj.2020.05.046 32867958 PMC7598119

[B19] Flock D. K. Preisinger R. (2007). Specialization and concentration as contributing factors to the success of the poultry industry in the global food market. Eur. Poult Sci. 71, 193–199. doi: 10.1016/S0003-9098(25)00544-2

[B20] Fulton J. E. (2006). Avian genetic stock preservation: an industry perspective. Poult Sci. 85, 227–231. doi: 10.1093/ps/85.2.227 16523618

[B21] Garant R. C. Tobalske B. W. Ben Sassi N. van Staaveren N. Tulpan D. Widowski T. . (2023). Does wing use and disuse cause behavioural and musculoskeletal changes in domestic fowl (Gallus gallus domesticus)? R. Soc Open Sci. 10, 220809. doi: 10.1098/rsos.220809 36704252 PMC9874265

[B22] Gebhardt-Henrich S. G. Fröhlich E. K. F. (2015). Early onset of laying and bumblefoot favor keel bone fractures. Animals 5, 1192–1206. doi: 10.3390/ani5040406 26633520 PMC4693210

[B23] Gebhardt-Henrich S. G. Pfulg A. Fröhlich E. K. F. Käppeli S. Guggisberg D. Liesegang A. . (2017). Limited associations between keel bone damage and bone properties measured with computer tomography, three-point bending test, and analysis of minerals in Swiss laying hens. Front. Vet. Sci. 4, 128. doi: 10.3389/fvets.2017.00128 28848740 PMC5554496

[B24] Gretarsson P. Søvik Å. Thøfner I. Moe R. O. Toftaker I. Kittelsen K. (2024). Fracture morphology and ossification process of the keel bone in modern laying hens based on radiographic imaging. PloS One 19, e0312878. doi: 10.1371/journal.pone.0312878 39471193 PMC11521247

[B25] Habig C. Henning M. Baulain U. Jansen S. Scholz A. M. Weigend S. (2021a). Keel bone damage in laying hens-its relation to bone mineral density, body growth rate and laying performance. Animals 11, 1546. doi: 10.3390/ani11061546 34070496 PMC8228274

[B26] Habig C. Weigend A. Baulain U. Petow S. Weigend S. (2021b). Influence of age and phylogenetic background on blood parameters associated with bone metabolism in laying hens. Front. Physiol. 12, 678054. doi: 10.3389/fphys.2021.678054 33995131 PMC8117343

[B27] Hanlon C. Takeshima K. Bédécarrats G. Y. (2021). Changes in the control of the hypothalamic-pituitary gonadal axis across three differentially selected strains of laying hens (Gallus gallus domesticus). Front. Physiol. 12, 651491. doi: 10.3389/fphys.2021.651491 33841186 PMC8027345

[B28] Hanlon C. Takeshima K. Kiarie E. G. Bédécarrats G. Y. (2022a). Bone and eggshell quality throughout an extended laying cycle in three strains of layers spanning 50 years of selection. Poult Sci. 101, 101672. doi: 10.1016/j.psj.2021.101672 35074590 PMC8789532

[B29] Hanlon C. Ziezold C. J. Bédécarrats G. Y. (2022b). The diverse roles of 17β-estradiol in non-gonadal tissues and its consequential impact on reproduction in laying and broiler breeder hens. Front. Physiol. 13, 942790. doi: 10.3389/fphys.2022.942790 35846017 PMC9283702

[B30] Hildebrand L. Gerloff C. Winkler B. Eusemann B. K. Kemper N. Petow S. (2024). Japanese quails (Coturnix japonica) show keel bone damage during the laying period-a radiography study. Front. Physiol. 15, 1368382. doi: 10.3389/fphys.2024.1368382 38545371 PMC10967949

[B31] Hildebrand L. Neukirchen S. Fellmin M. Mehlhorn J. Petow S. Schrader L. . (2026). Comparison of keel bone traits, eggshell production, and physiological parameters between a hybrid layer and two low-performing chicken genotypes. Front. Physiol. 17, 1782139. doi: 10.3389/fphys.2026.1782139 41907534 PMC13021905

[B32] Jansen S. Baulain U. Habig C. Weigend A. Halle I. Scholz A. M. . (2020). Relationship between bone stability and egg production in genetically divergent chicken layer lines. Animals 10, 850. doi: 10.3390/ani10050850 32423072 PMC7278460

[B33] Jung L. Hillemacher S. Tiemann I. Lepke M. Hinrichs D. (2024). Presence of keel bone damage in laying hens, pullets and roosters of local chicken breeds. PloS One 19, e0297586. doi: 10.1371/journal.pone.0297586 38277352 PMC10817119

[B34] Jung L. Niebuhr K. Hinrichsen L. K. Gunnarsson S. Brenninkmeyer C. Bestman M. . (2019). Possible risk factors for keel bone damage in organic laying hens. Animal 13, 2356–2364. doi: 10.1017/S175173111900003X 30808429

[B35] Käppeli S. Gebhardt-Henrich S. G. Fröhlich E. Pfulg A. Stoffel M. H. (2011). Prevalence of keel bone deformities in Swiss laying hens. Br. Poult Sci. 52, 531–536. doi: 10.1080/00071668.2011.615059 22029778

[B36] Kittelsen K. E. Gretarsson P. Jensen P. Christensen J. P. Toftaker I. Moe R. O. . (2021). Keel bone fractures are more prevalent in White Leghorn hens than in Red Jungle fowl hens-a pilot study. PloS One 16, e0255234. doi: 10.1371/journal.pone.0255234 34314465 PMC8315525

[B37] Kittelsen K. E. Moe R. O. Hansen T. B. Toftaker I. Christensen J. P. Vasdal G. (2020). A descriptive study of keel bone fractures in hens and roosters from four non-commercial laying breeds housed in furnished cages. Animals 10, 2192. doi: 10.3390/ani10112192 33238563 PMC7700571

[B38] Kolakshyapati M. Flavel R. J. Sibanda T. Z. Schneider D. Welch M. C. Ruhnke I. (2019). Various bone parameters are positively correlated with hen body weight while range access has no beneficial effect on tibia health of free-range layers. Poult Sci. 98, 6241–6250. doi: 10.3382/ps/pez487 31504903 PMC8913749

[B39] Kwakkel R. P. van Esch J. A. Ducro B. J. Koops W. J. (1995). Onset of lay related to multiphasic growth and body composition in White Leghorn pullets provided ad libitum and restricted diets. Poult Sci. 74, 821–832. doi: 10.3382/ps.0740821 7603959

[B40] Leszczynski D. E. Hagan R. C. Bitgood J. J. Kummerow F. A. (1985). Relationship of plasma estradiol and progesterone levels to egg productivity in domestic chicken hens. Poult Sci. 64, 545–549. doi: 10.3382/ps.0640545 3991427

[B41] Lewis P. D. Perry G. C. Morris T. R. (1997). Effect of size and timing of photoperiod increase on age at first egg and subsequent performance of two breeds of laying hen. Br. Poult Sci. 38, 142–150. doi: 10.1080/00071669708417959 9158887

[B42] LOHMANN BREEDERS GmbH (2021a). Lohmann Brown-Classic: Leistungsdaten Körpergewichtstabellen. Available online at: https://lohmann-deutschland.de/wp-content/uploads/2025/01/Protected_LB_Zusatzheft_Alternative-Haltung_LB-Classic_DE_06.21_V01-21_high.pdf (Accessed September 8, 2026).

[B43] LOHMANN BREEDERS GmbH (2021b). Lohmann Sandy: Leistungsdaten Körpergewichtstabellen. Available online at: https://lohmann-deutschland.de/wp-content/uploads/2025/01/Protected_LB_Zusatzheft_Alternative-Haltung_Sandy_DE_06.21_V01-21_high.pdf (Accessed September 8, 2026).

[B44] Malomane D. K. Simianer H. Weigend A. Reimer C. Schmitt A. O. Weigend S. (2019). The SYNBREED chicken diversity panel: a global resource to assess chicken diversity at high genomic resolution. BMC Genomics 20, 345. doi: 10.1186/s12864-019-5727-9 31064348 PMC6505202

[B45] Mans C. Taylor W. M. (2008). Update on neuroendocrine regulation and medical intervention of reproduction in birds. Vet. Clin. North Am. Exot Anim Pract. 11, 83–105. doi: 10.1016/j.cvex.2007.09.003 18165139

[B46] Mehlhorn J. Höhne A. Baulain U. Schrader L. Weigend S. Petow S. (2022). Estradiol-17ß is influenced by age, housing system, and laying performance in genetically divergent laying hens (Gallus gallus f.d.). Front. Physiol. 13. doi: 10.3389/fphys.2022.954399 35936910 PMC9353941

[B47] Montalcini C. M. Toscano M. J. Asher L. Petelle M. B. (2024). Keel bone fractures affect laying hens' mobility, but no evidence for reciprocal effects. PloS One 19, e0306384. doi: 10.1371/journal.pone.0306384 38968298 PMC11226069

[B48] Morris T. R. Sharp P. J. Butler E. A. (1995). A test for photorefractoriness in high-producing stocks of laying pullets. Br. Poult Sci. 36, 763–769. doi: 10.1080/00071669508417820 8746977

[B49] Nasr M. A. F. Murrell J. Nicol C. J. (2013). The effect of keel fractures on egg production, feed and water consumption in individual laying hens. Br. Poult Sci. 54, 165–170. doi: 10.1080/00071668.2013.767437 23647178

[B50] Nasr M. A. F. Nicol C. J. Murrell J. (2012). Do laying hens with keel bone fractures experience pain? PloS One 7, e42420. doi: 10.1371/journal.pone.0042420.g001 22927930 PMC3425496

[B51] Nielsen S. S. Alvarez J. Bicout D. J. Calistri P. Canali E. Drewe J. A. . (2023). Scientific Opinion on the welfare of laying hens on farm. EFSA Journal 21, 7789. doi: 10.2903/j.efsa.2023.7789

[B52] Okumura J. Mori N. Muramatsu T. Tasaki I. Saito F. (1988). Analysis of factors affecting year-round performance of Single Comb White Leghorn laying hens reared under an open-sided housing system. Poult Sci. 67, 1130–1138. doi: 10.3382/ps.0671130 3217304

[B53] Onagbesan O. M. Metayer S. Tona K. Williams J. Decuypere E. Bruggeman V. (2006). Effects of genotype and feed allowance on plasma luteinizing hormones, follicle-stimulating hormones, progesterone, estradiol levels, follicle differentiation, and egg production rates of broiler breeder hens. Poult Sci. 85, 1245–1258. doi: 10.1093/ps/85.7.1245 16830866

[B54] Parsons A. H. Combs G. F. (1981). Blood ionized calcium cycles in the chicken. Poult Sci. 60, 1520–1524. doi: 10.3382/ps.0601520 7198779

[B55] Petrik M. T. Guerin M. T. Widowski T. M. (2015). On-farm comparison of keel fracture prevalence and other welfare indicators in conventional cage and floor-housed laying hens in Ontario, Canada. Poult Sci. 94, 579–585. doi: 10.3382/ps/pev039 25713398

[B56] Prastiya R. A. Madyawati S. P. Sari S. Y. Nugroho A. P. (2022). Effect of follicle-stimulating hormone and luteinizing hormone levels on egg-laying frequency in hens. Vet. World 15, 2890–2895. doi: 10.14202/vetworld.2022.2890-2895 36718318 PMC9880839

[B57] Pulcini D. Mattioli S. Angelucci E. Chenggang W. Cartoni Mancinelli A. Napolitano R. . (2023). Shape and fractures of carina sterni in chicken genotypes with different egg deposition rates reared indoor or free-range. Sci. Rep. 13, 22495. doi: 10.1038/s41598-023-49909-1 38110659 PMC10728074

[B58] Qasir H. Reyer H. Oster M. Ponsuksili S. Trakooljul N. Sommerfeld V. . (2025). Effects of a transient lack of dietary mineral phosphorus on renal gene expression and plasma metabolites in two high-yielding laying hen strains. BMC Genomics 26, 129. doi: 10.1186/s12864-025-11294-6 39930376 PMC11812262

[B59] Qin H. Jiao W. (2022). Correlation of muscle mass and bone mineral density in the NHANES US general population 2017-2018. Medicine 101, e30735. doi: 10.1097/MD.0000000000030735 36181112 PMC9524880

[B60] Rentsch A. K. Ross E. Harlander A. Niel L. Siegford J. M. Widowski T. M. (2023). The development of laying hen locomotion in 3D space is affected by early environmental complexity and genetic strain. Sci. Rep. 13, 10084. doi: 10.1038/s41598-023-35956-1 37344513 PMC10284819

[B61] Riber A. B. Casey-Trott T. M. Herskin M. S. (2018). The influence of keel bone damage on welfare of laying hens. Front. Vet. Sci. 5, 6. doi: 10.3389/fvets.2018.00006 29541640 PMC5835507

[B62] Rufener C. Baur S. Stratmann A. Toscano M. J. (2019). Keel bone fractures affect egg laying performance but not egg quality in laying hens housed in a commercial aviary system. Poult Sci. 98, 1589–1600. doi: 10.3382/ps/pey544 30481360

[B63] Sallam M. Göransson L. Larsen A. Alhamid W. Johnsson M. Wall H. . (2024). Comparisons of longitudinal radiographic measures of keel bones, tibiotarsal bones, and pelvic bones versus post-mortem measures of keel bone damage in Bovans Brown laying hens housed in an aviary system. Front. Vet. Sci. 11, 1432665. doi: 10.3389/fvets.2024.1432665 39403210 PMC11472762

[B64] Sallam M. Göransson L. Larsen A. Wall H. Alhamid W. Gunnarsson S. . (2025). Genetics of digital phenotypes of keel bone in layer chickens and correlations with keel bone fractures and deviations. Genet. Sel Evol. 57, 69. doi: 10.1186/s12711-025-01016-7 41310424 PMC12661725

[B65] Sandilands V. Moinard C. Sparks N. H. C. (2009). Providing laying hens with perches: fulfilling behavioural needs but causing injury? Br. Poult Sci. 50, 395–406. doi: 10.1080/00071660903110844 19735008

[B66] Schmidt H. (1999). Handbuch Rasse- Und Ziergeflügel: Hühner Und Zwerghühner (Stuttgart: Ulmer).

[B67] Silverin B. Kikuchi M. Ishii S. (1997). Seasonal changes in follicle-stimulating hormone in free-living great tits. Gen. Comp. Endocrinol. 108, 366–372. doi: 10.1006/gcen.1997.6980 9405113

[B68] Sinclair-Black M. Garcia R. A. Ellestad L. E. (2023). Physiological regulation of calcium and phosphorus utilization in laying hens. Front. Physiol. 14, 1112499. doi: 10.3389/fphys.2023.1112499 36824471 PMC9942826

[B69] Squire M. E. Veglia M. K. Drucker K. A. Brazeal K. R. Hahn T. P. Watts H. E. (2017). Estrogen levels influence medullary bone quantity and density in female house finches and pine siskins. Gen. Comp. Endocrinol. 246, 249–257. doi: 10.1016/j.ygcen.2016.12.015 28039066 PMC5392170

[B70] Stratmann A. Fröhlich E. K. F. Gebhardt-Henrich S. G. Harlander-Matauschek A. Würbel H. Toscano M. J. (2015). Modification of aviary design reduces incidence of falls, collisions and keel bone damage in laying hens. Appl. Anim Behav. Sci. 165, 112–123. doi: 10.1016/j.applanim.2015.01.01242574925

[B71] Tanabe Y. Nakamura T. Tanase H. Doi O. (1981). Comparisons of plasma LH, progesterone, testosterone and estradiol concentrations in male and female chickens (Gallus domesticus) from 28 to 1141 days of age. Endocrinol. Jpn. 28, 605–613. doi: 10.1507/endocrj1954.28.605 7344950

[B72] Thiruvenkadan A. K. Panneerselvam S. Prabakaran R. (2010). Layer breeding strategies: an overview. Worlds Poult Sci. J. 66, 477–502. doi: 10.1017/S004393391000055341292463

[B73] Thøfner I. Dahl J. Christensen J. P. (2021). Keel bone fractures in Danish laying hens: Prevalence and risk factors. PloS One 16, e0256105. doi: 10.1371/journal.pone.0256105 34388183 PMC8362975

[B74] Thøfner I. Hougen H. P. Villa C. Lynnerup N. Christensen J. P. (2020). Pathological characterization of keel bone fractures in laying hens does not support external trauma as the underlying cause. PloS One 15, e0229735. doi: 10.1371/journal.pone.0229735 32150551 PMC7062247

[B75] Tracy L. M. Temple S. M. Bennett D. C. Sprayberry K. A. Makagon M. M. Blatchford R. A. (2019). The reliability and accuracy of palpation, radiography, and sonography for the detection of keel bone damage. Animals 9, 894. doi: 10.3390/ani9110894 31683826 PMC6912489

[B76] Visser H. K. (1973). Some physiological and clinical aspects of puberty. Arch. Dis. Child 48, 169–182. doi: 10.1136/adc.48.3.169 4571396 PMC1648094

[B77] Wan Y. Ma R. Khalid A. Chai L. Qi R. Liu W. . (2021). Effect of the pellet and mash feed forms on the productive performance, egg quality, nutrient metabolism, and intestinal morphology of two laying hen breeds. Animals 11, 701. doi: 10.3390/ani11030701 33807718 PMC7998960

[B78] Warren D. C. (1937). Physiologic and genetic studies of crooked keels in chicken. Kans Tech Bull. 44, 2–32.

[B79] Wei H. Bi Y. Xin H. Pan L. Liu R. Li X. . (2020). Keel fracture changed the behavior and reduced the welfare, production performance, and egg quality in laying hens housed individually in furnished cages. Poult Sci. 99, 3334–3342. doi: 10.1016/j.psj.2020.04.001 32616227 PMC7597854

[B80] Wei H. Chen Y. Nian H. Wang J. Liu Y. Yang K. . (2021). Abnormal bone metabolism may be a primary causative factor of keel bone fractures in laying hens. Animals 11, 3133. doi: 10.3390/ani11113133 34827866 PMC8614394

[B81] Whitehead C. C. (2004). Overview of bone biology in the egg-laying hen. Poult Sci. 83, 193–199. doi: 10.1093/ps/83.2.193 14979569

[B82] Whitehead C. C. Fleming R. H. (2000). Osteoporosis in cage layers. Poult Sci. 79, 1033–1041. doi: 10.1093/ps/79.7.1033 10901207

[B83] Wilkins L. J. McKinstry J. L. Avery N. C. Knowles T. G. Brown S. N. Tarlton J. . (2011). Influence of housing system and design on bone strength and keel bone fractures in laying hens. Vet. Rec 169, 414. doi: 10.1136/vr.d4831 21862469

[B84] Wilson S. Duff S. R. I. (1990). Morphology of medullary bone during the egg formation cycle. Res. Vet. Sci. 48, 216–220. doi: 10.1016/S0034-5288(18)30993-7 2333428

[B85] Wilson S. Thorp B. H. (1998). Estrogen and cancellous bone loss in the fowl. Calcif Tissue Int. 62, 506–511. doi: 10.1007/s002239900470 9576978

[B86] Yang H. M. Wang Y. Wang Z. Y. Wang X. X. (2017). Seasonal and photoperiodic regulation of reproductive hormones and related genes in Yangzhou geese. Poult Sci. 96, 486–490. doi: 10.3382/ps/pew340 28123085

[B87] Ziomkiewicz A. Ellison P. T. Lipson S. F. Thune I. Jasienska G. (2008). Body fat, energy balance and estradiol levels: a study based on hormonal profiles from complete menstrual cycles. Hum. Reprod. 23, 2555–2563. doi: 10.1093/humrep/den213 18641044

